# A Comprehensive Review of Semiconductor Ultraviolet Photodetectors: From Thin Film to One-Dimensional Nanostructures

**DOI:** 10.3390/s130810482

**Published:** 2013-08-13

**Authors:** Liwen Sang, Meiyong Liao, Masatomo Sumiya

**Affiliations:** 1 International Center for Young Scientists (ICYS), National Institute for Materials Science (NIMS), 1-1 Namiki, Tsukuba, Ibaraki 305-0044, Japan; 2 JST-PRESTO, the Japan Science and Technology Agency, Tokyo 102-0076, Japan; 3 Optical and Electronic Materials Unit, National Institute for Materials Science (NIMS), Tsukuba, Ibaraki 305-0044, Japan; E-Mails: meiyong.liao@nims.go.jp (M.L.); sumiya.masatomo@nims.go.jp (M.S.); 4 JST-ALCA, the Japan Science and Technology Agency, Tokyo 102-0076, Japan

**Keywords:** ultraviolet photodetector, semiconductor, thin film, one-dimensional nanostructures

## Abstract

Ultraviolet (UV) photodetectors have drawn extensive attention owing to their applications in industrial, environmental and even biological fields. Compared to UV-enhanced Si photodetectors, a new generation of wide bandgap semiconductors, such as (Al, In) GaN, diamond, and SiC, have the advantages of high responsivity, high thermal stability, robust radiation hardness and high response speed. On the other hand, one-dimensional (1D) nanostructure semiconductors with a wide bandgap, such as *β*-Ga_2_O_3_, GaN, ZnO, or other metal-oxide nanostructures, also show their potential for high-efficiency UV photodetection. In some cases such as flame detection, high-temperature thermally stable detectors with high performance are required. This article provides a comprehensive review on the state-of-the-art research activities in the UV photodetection field, including not only semiconductor thin films, but also 1D nanostructured materials, which are attracting more and more attention in the detection field. A special focus is given on the thermal stability of the developed devices, which is one of the key characteristics for the real applications.

## 1. Introduction

Photodetection in the ultraviolet (UV) region has drawn extensive attention owing to its various applications in industry, instrument, and our daily life. UV light is typically divided into four spectral regions: UV-A (for wavelengths between 400 and 320 nm), UV-B (for wavelengths between 320 and 280 nm), UV-C (for wavelengths between 280 and 200 nm), and far UV (for wavelength between 200 and 10 nm, which reaches the X-ray spectral low energy frontier) [1–3]. Most of the UV light from the Sun is absorbed by the atmospheric ozone layer. Solar radiation with wavelength longers than 280 nm can penetrate the atmosphere and reach the Earth. For this reason, UV detectors that have high sensitivity to UV-C and far UV radiation compared to radiation with wavelength longer than 280 nm can be called ‘solar-blind’. As visible light covers the range of wavelengths from 400 to 700 nm, UV detectors that have high sensitivity to UV-A, UV-B, UV-C and far UV radiation compared to radiation with wavelengths longer than 400 nm are called ‘visible-blind’. The detection of UV radiation presents a wide range of applications, such as chemical, environmental and biological analysis or monitoring, flame and radiation detection, astronomical studies, and optical communications. A high-performance photodetector should satisfy the *5S* requirements of high *sensitivity*, high *signal-to-noise ratio*, high spectral *selectivity*, high *speed*, and high *stability* [4]. In some cases such as the flame detection for a hot engine, high performance thermally stable detectors are required.

The well-established UV-enhanced Si technology has some limitations in the UV detection. Since the bandgap energy of Si is 1.1 eV, costly high pass optical filters and phosphors are needed to stop low energy photons. Therefore, the device performance is degraded with temperature, as evidenced by lower efficiency and increasing dark currents. The development of wide-bandgap-semiconductor UV photodetectors has now emerged, such as GaN-based system, diamond, or SiC-based system, which do not need the insertion of filters, showing their potential for high-temperature applications [5–7]. Recently, we found that the detectors can work at the temperature up to 523 K by using calcium fluoride (CaF_2_) as the insulation layer in a InGaN-based metal-insulator-semiconductor (MIS) Schottky-type photodiodes [8]. On the other hand, one-dimensional (1D) nanostructured UV detectors are attracting more and more attention owing to the improved sensitivity to light due to their large surface-to-volume ratio and Debye length comparable to their small size [9]. An enhanced photosensitivity has been observed in 1D nanostructured UV detectors, such as ZnO nanowires, Ga_2_O_3_ nanowires and nanobelts, GaN nanowires, or other metal-oxide nanostructures [10–13]. However, as a result of the surface dominated photocurrent transport, the high-temperature UV detection based on the nanostructured semiconductors are facing predicaments. To resolve this bottleneck, bulk-dominated photocurrent dynamics are preferred.

In this article, we provide a comprehensive review of the state-of-the-art research activities on the semiconductor UV photodetectors, not just concentrated on semiconductor thin films, but also with a special emphasis on 1D nanostructures, highlighting our recent progress in the area of high-performance UV photodetectors based on *β*-Ga_2_O_3_ nanowires working at temperatures as high as 553 K with high detectivity. We also present recently proposed concepts such as hybrid- and hetero-integration techniques in the photodetection field.

## 2. Semiconductor Thin-Film UV Photodetector

### 2.1. III-Nitride Semiconductor System

Group-III nitrides (AlN, GaN, InN, and their ternary and quaternary compounds) are considered to be a strategic technology for the development of high-performance UV photodetectors. They have many advantages such as the ideal spectral selectivity with wide direct bandgaps from the deep UV to the infrared region, high breakdown field, high thermal stability, radiation hardness, and expected high responsivity [14]. The saturation velocity of GaN is higher than that of GaAs, and the optical photon energies are about three times larger, which favors transient transport by delaying the onset of optical photon emission. III-Nitride semiconductors including GaN (360 nm with the bandgap of 3.42 eV), In_x_Ga_1-x_N (bandgap assigned today to InN is 0.65 eV), and Al_x_Ga_1-x_N (bandgap of AlN is 6.2 eV with 210 nm), provide the full UV region photodetections [15,16]. The visible-blind with wavelengths lower than 400 nm can be reached using In_0.1_Ga_0.9_N alloys. Al_x_Ga_1-x_N alloys with *x* higher than 40% are considered to be among the best candidates for solar-blind detection. Besides, the high chemical and thermal stability of GaN-based detectors have increasingly attracted fundamental research interest in high-temperature and high photon energy detection, or even for high energy particle detection [17].

**Figure 1.**
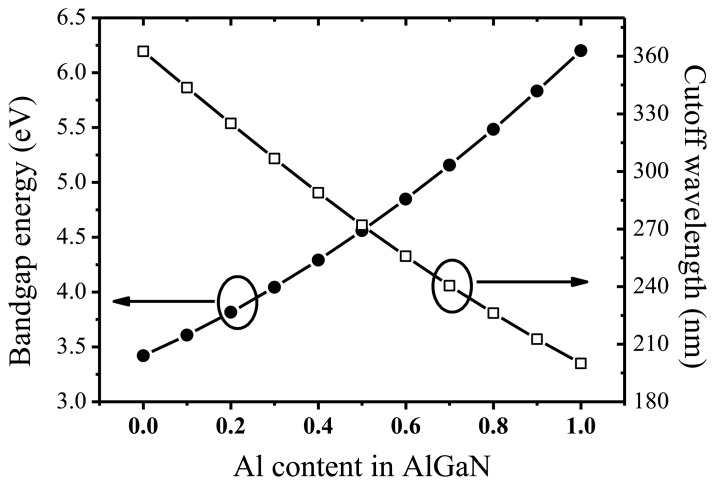
Bandgap and cutoff wavelength of AlGaN dependent on the Al mole fraction.

The study of photoconductive properties of GaN was started by Pankove and Berkeyheiser in 1974 [18]. With the breakthrough of the epitaxy technique for GaN film by metal-organic chemical vapor deposition (MOCVD) in the 1990s, the first GaN-based UV photoconductor was developed by Khan *et al*. in 1992 [19]. Since then, various teams have been exploring and developing different types of photodetector structures, such as photoconductor, metal-semiconductor-metal (MSM), Schottky barrier, metal-insulator-semiconductor (MIS), *p-n* junction, and *p-i-n* junction (Figure 1). The first stage focused on the development of high responsivity, high speed, true visible-blind using GaN and solar-blind with AlGaN (high Al mole fractions) photodetectors, which are strongly related with the film epitaxy techniques. A second stage was committed to develop the real applications such as focal-plane arrays solar-blind photodetection. These devices have been introduced and summarized by numerous review papers and book chapters [2,4,7,20]. Thus, in this paper, we limit our discussion to the state-of-the-art of III-nitride (In,Ga)N-based visible-blind and (Al,Ga)N-based solar-blind photodetectors. In addition, a special focus on the high-temperature stability of nitride-based detectors will be included in Section 2.6.

#### 2.1.1. (Al, Ga)N-Based Photodetectors

Due to the interesting applications, including early missile threat detection and interception, chemical and biological threat detection, UV flame monitoring, UV environmental monitoring, *etc*., there have been tremendous developments in the field of solar-blind photodetectors with a cut-off response lower than 280 nm using AlGaN since 1999.

Figure 1 shows the relationship of bandgap energy (cutoff wavelength) of Al_x_Ga_1-x_N semiconductor with Al mole fraction. As can be seen, to realize solar-blind detection, the Al composition in Al_x_Ga_1-x_N should be higher than 40%. High-quality AlGaN films are the key element in obtaining high-performance photodetectors. Compared to GaN, the growth of AlGaN especially with a high Al amount has proved to be significantly difficult, which lies in the lower migration of Al atoms than that of Ga atoms, and strong parasitic reactions. Therefore, the layer-by-layer growth with the atomic steps on the surface for the films is difficult to achieve compared with the well-developed GaN template. As a result, high-density defects, such as dislocations, grain boundaries, or stacking faults, are much easier to generate [21].

Previously, AlGaN films were usually deposited on GaN templates to fabricate UV devices. However, as a result of the tensile strain from the smaller in-plane lattice constant of AlN compared to that of GaN, cracks were generated when the thickness of the AlGaN film was higher than its critical thickness. On the other hand, to avoid the optical absorption from contacts, back-illuminated configurations are needed for the Schottky or *p-i-n* type photodetectors. Nevertheless, most of the UV light was absorbed by the narrow-bandgap GaN template. AlGaN films grown on sapphire consist of many fine mosaic blocks, which also result in the cracks due to the coalescence of these blocks. The cracks result in a high dark current of μA or mA, a low rejection ratio, and restrict the performance of the UV devices. The highest Al composition without cracks obtained on GaN template is ∼30% by using the interlayer technique [22]. The best performance of the AlGaN photodetectors on GaN template used the Schottky structure, and showed a dark current of 9 nA at −5 V, cutoff wavelength of 310 nm, and UV/visible discrimination ratio of 10^4^.

To realize the real solar-blind detection using Al-rich AlGaN film, AlN is the best choice as the template to avoid the cracks and absorption losses. However, the development of an AlN epilayer was restricted for a long time due to the temperature limitations in the conventional MOCVD system. Khan *et al*. suggested the use of the pulse atomic layer deposition (PALE) approach to grow AlN at a relatively lower temperature, in which, the group III and group V precursors are sequentially modulated to increase the migration of Al atoms and avoid any parasitic reactions [23]. Recently, a modified PALE approach called migration-enhanced MOCVD (MEMOCVD) was employed to further improve the surface and crystalline quality of AlN and AlGaN [24]. Furthermore, it was reported that the AlN/AlGaN superlatices can be used to alleviate the tensile strain and avoid the crack problems in AlGaN thick films with a high amount of Al [25]. AlGaN films deposited using MEMOCVD and AlN/AlGaN superlattices were also determined to be of superior quality, in terms of structural quality and minority carrier lifetime [26]. With the improvement of the film quality of AlGaN, the performance of solar-blind photodetectors was also improved.

**Figure 2.**
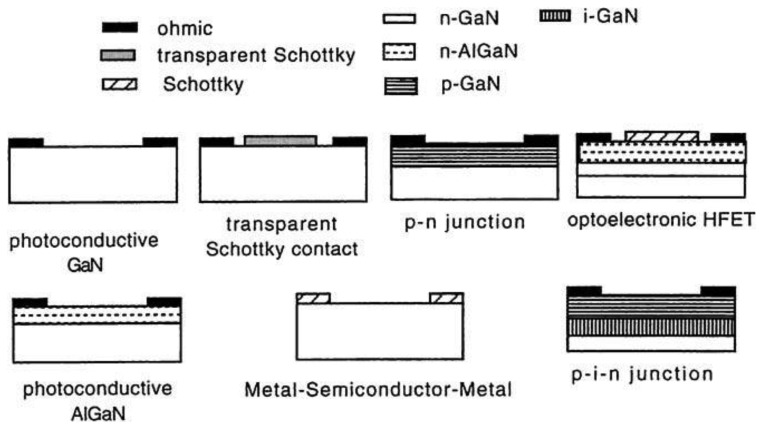
Schematic photodetector structures based on III-Nitride semiconductors. (Reprinted from Reference [27]).

Some of the basic photodetector structures have been fabricated using AlGaN layers (Figure 2). AlGaN-based photoconductors have been investigated in detail by a number of groups. They typically showed a high responsivity (>100A/W) [28–31], but a strong persistent photoconductivity (PPC), which means the photocurrent persists for a long time (hours) after the light is shut off [32,33]. The measured responsivity is determined not just by the optical absorption in the semiconductor, but also by the time that the sample has been kept in the dark. The response speed for the photoconductor is very slow, which is not suitable for the real applications. Similar to the photoconductor, symmetrical MSM-type photodiodes are also not able to work at zero bias. They use two back-to-back Schottky contacts. Early GaN-based MSM detectors demonstrated a high quantum efficiency at the high applied voltage, which showed the existence of photocurrent gain [34–36]. AlGaN-based solar-blind MSM detectors have also been reported with quantum efficiency more than 40% at 262 nm [37–40]. However, due to its bias-driven nature, noise is the biggest problem in this kind of photodetectors.

**Figure 3.**
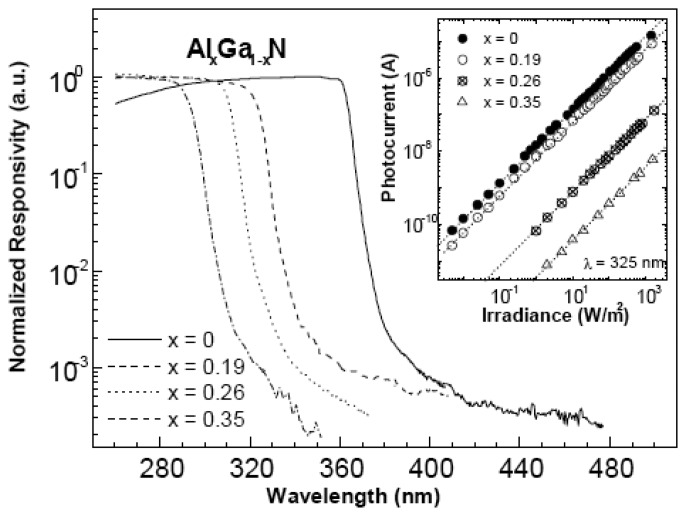
Spectral response of AlGaN-based Schottky barrier photodiodes for different Al mole fraction. (Reprinted from Reference [7]).

The Schottky-type photodetectors illustrate a fast response speed and little PPC effect. Metals with high work function, such as Pt, Ni, Pd or Au, are commonly used for the Schottky contact on AlGaN. To obtain the sufficient optical absorption, the metals should be thin enough for transmittance. The Schottky barrier GaN photodetectors were first reported by Khan *et al*., who demonstrated a Ti Schottky diode on *p*-type GaN, with a UV/visible ratio of more than two orders of magnitude [41]. Chen *et al*. reported a 50 Å Pd Schottky barrier detector on *n*-GaN, with a responsivity of 180 mA/W, and resistance-capacitance (RC)-limited constant of 118 ns [42]. The first AlGaN-based photodiode using 50 Å Pd Schottky barriers with an Al content of 26% was reported by the same group. It showed a responsivity of 70 mA/W and a minimum time constant of 1.6 μs [43]. An enhancement of the Schottky barrier height is observed as Al composition increased, which is resulted from the decrease in the electron affinity of the semiconductors [44]. Figure 3 shows the spectral responsivity of AlGaN Schottky diodes with different Al contents.

**Figure 4.**
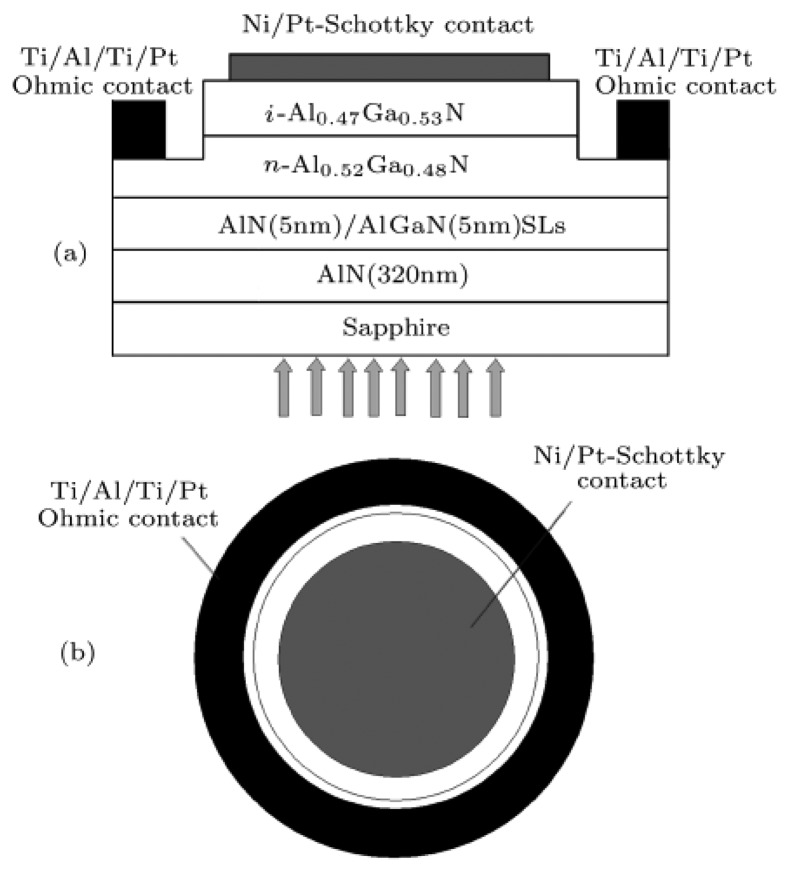
(**a**) Structure of Schottky-type back-illuminated photodetectors; (**b**) plan view of the contacts. (Reprinted from Reference [47]).

The cutoff wavelength is decreasing from 362 to 293 nm by increasing Al composition. The UV/visible discrimination ratio is more than three orders of magnitude. The absolute responsivity is decreased with Al content increasing, which is 54, 45, 30, and 10 mA/W for *x* = 0, 0.19, 0.26, and 0.35, respectively. To obtain the sufficient optical absorption, the metals should be thin enough for transmittance. However, the best transmittance for the reported metal Au or Ni/Au bilayers still has a light loss of ∼30% in the 350–50 nm spectral range [45,46]. Moreover, thin metal films are usually not uniform, and lead to a high leakage current. The external quantum efficiency (EQE) is also reduced by the semi-transparent Schottky contact. Back-illuminated Schottky barrier photodetectors can overcome these drawbacks. To avoid the absorption from GaN template, the back-illuminated AlGaN-based Schottky photodetectors were usually deposited on sapphire or AlN template. Figure 4 shows the structure of the Schottky-type back-illuminated photodetectors, and the contact configuration [47]. The cutoff wavelength is from 280 to 292 nm with different Al composition without bias. The devices all displayed a low dark current, a high breakdown voltage more than 40 V, and a specific detectivity of more than 3.3 × 10^12^ cmHz^1/2^W^−1^.

P-I-N type detectors offer low bias voltage, low dark currents, and high speed. With the development of doping in *p*-GaN and *p*-AlGaN, there are more and more reports on the AlGaN-based *p-i-n* structured photodetectors. Biyikli *et al*. reported on an AlGaN-based *p-i-n* type photodetector with a cutoff wavelength of 283 nm, a responsivity of 0.11 A/W biased at −10 V, and specific detectivity of 4.9 × 10^14^ cmHz^1/2^W^−1^[48]. The structure optimization is important to improve the performance of the *p-i-n* detectors. P-AlGaN/GaN superlattices were used to improve the light absorption and reduce the serial resistance [49]. In 2003, the Al-rich Al_0.6_Ga_0.4_N is used to be the window layer, and a high external quantum efficiency of 58% was obtained at 274 nm without bias. The responsivity is 0.13 A/W, with an external quantum efficiency of 57%. Lambert *et al*. used back-illuminated structured detectors, and obtained the low dark current densities, with a high external quantum efficiency of 35% at 280 nm [50]. With the development of AlN template, high-performance AlGaN-based solar-blind photodetectors were developed. Collins *et al*. obtained the EQE of 42% at 269 nm, with a specific detectivity of 2.0 × 10^14^ cmHz^1/2^W^−1^[51]. A high quantum efficiency of 72% under 5 V was obtained by using the back-illuminated structure on high-quality AlN template, with Al_0.87_Ga_0.13_N/AlN superlattices train-relief layer, and a highly conductive Si-In co-doped Al_0.5_Ga_0.5_N contact layer. The solar-blind photodetector showed a peak responsivity of 136 mA/W at 282 nm [52].

#### 2.1.2. (In, Ga)N-Based Visible-Blind Photodetectors

In_x_Ga_1-x_N alloys with an In composition lower than 10% can be used for the UV-A visible-blind (320–400 nm) detection. Currently, Si-based photodetectors are widely used to detect optical energy in this region, but the performance of these devices is inadequate due to their narrow bandgaps. An InGaN-based photodetector, with its tunable direct bandgap energy, high theoretical responsivity, high breakdown voltage, and sharp cutoff wavelength, offers an alternative and potentially better approach for detecting UV-A light [53]. However, InGaN-based devices often suffer from a high leakage current, low discrimination ratio between UV and visible light, and a strong PPC due to high-density threading dislocations. Different from light emitting diodes, the active region of InGaN-based photodetectors should be thick enough to obtain sufficient light absorption.

InGaN/GaN multiple quantum wells (MQWs) were utilized by many groups to obtain a highly crystalline quality to overcome the technological limitations imposed by InGaN thin films. Despite the deficiencies in the material and contact quality, these structures offer remarkable advantages such as a lower expected noise, a more abrupt response cutoff, and an easier processing integration [54–56]. Rivera *et al*. fabricated Schottky-type photodetectors from InGaN/GaN MQWs with the In composition lower than 14%. The devices showed a responsivity of 0.06∼1 A/W with different In compositions and bias voltages. The UV/visible discrimination ratio was more than 10^4^. The cutoff wavelength was variable from 400 to 360 nm with depending on the In composition and polarization effect in the QWs structures. They also found that the increase in the number of QWs led to a higher responsivity due to the higher absorption. The MQWs were also utilized into the *p-n* junction structures. Chiou *et al*. developed InGaN/GaN MQW *p-n* junction photodetectors using a semi-transparent Ni/Au electrode [57,58]. The device showed a 20 V breakdown voltage and more than 10^5^ UV/visible discrimination ratio. The maximum responsivity was 1.76 A/W at 3 V at around 380 nm. It was also found the low frequency noise of the photodiodes was dominant by the 1/f type noise. High-quality InGaN thick films are the best choice to fabricate photodetectors due to the excess absorption and carrier transportation. The lattice-matched InGaN-based *p-i-n* structures lead to the improvement in the device performance [59]. This approach produced photodiodes with zero-bias responsivities up to 0.037 A/W at 426 nm. The peak responsivity wavelength ranged between 416 and 466 nm. However, the responsivity was decreased to 0.0035 A/W at 466 nm due to a degraded quality by increasing In composition. Su *et al*. achieved an EQE as high as 67% at 380 nm by using a 300 nm-thick In_0.11_Ga_0.89_N *p-i-n* photodetector [53].

It is noted that a large dark current existed for the InGaN-based photodetectors, especially for those with a high In composition. AlthoughChang *et al*. demonstrated seven orders of magnitude decrease of dark current by using 88 nm SiO_2_ insulation layer on InGaN/GaN MQW detectors [60]. Chen *et al*. and Zhou *et al*. also demonstrated two to three orders of magnitude decrease of leakage current by using Si_3_N_4_ layer, and lead to a UV/visible ratio more than 10^3^[61]. Chang *et al*. and Yu *et al*. reported a six orders of magnitude reduction of dark current by using unactivated Mg-doped *p*-GaN for MIS photodetectors [62,63]. However, none of these detectors showed time response performance. Table 1 summaries the performance of the InGaN-based photodetectors using different insulation layers.

**Figure 5.**
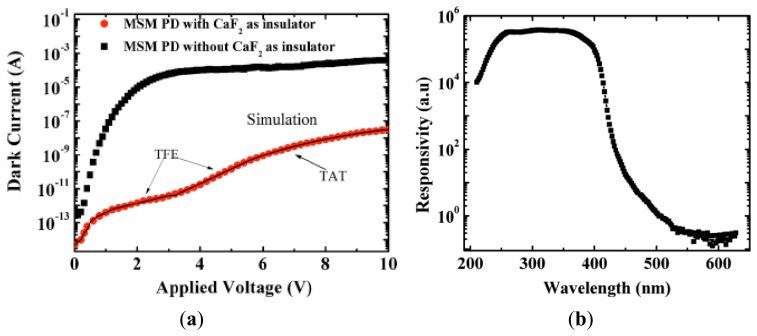
(**a**) The typical dark *I-V* characteristics for MSM photodetector without and with CaF_2_ insulator; (**b**) Photocurrent spectra measured under the illumination of xenon lamp at the applied voltage of 1 V. (Reprinted from Reference [64]).

**Figure 6.**
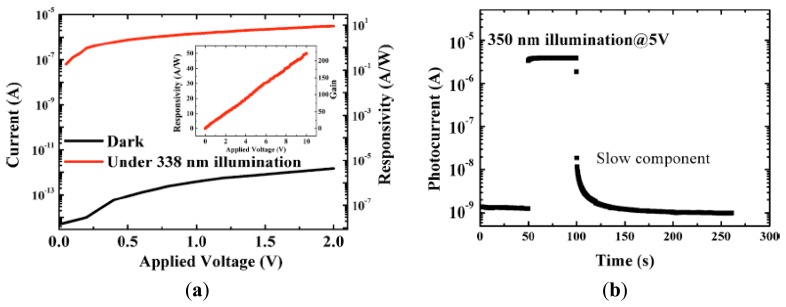
(**a**) *I-V* characteristics and responsivity in the dark and upon 338 nm light illumination. The inset shows the responsivity dependence on the applied voltage; (**b**) Time response upon 350 nm light illumination measured by a mechanical chopping method. (Reprinted from Reference [64]).

We proposed to use calcium fluoride (CaF_2_) with a super-wide bandgap (12 eV) as the insulation layer between the Schottky contact and a high-quality InGaN film in the MSM structured photodetectors [64]. Compared with the normally used SiO_2_ insulator, besides a wider bandgap, CaF_2_ exhibits a larger effective mass of carriers, a high electric strength, high thermal conductivity, and radiation robustness. By using 5 nm-thick CaF_2_ insulator, the dark current is drastically reduced by six orders of magnitude compared with those without CaF_2_, resulting in an extremely high discrimination ratio larger than 10^6^ between UV and visible light, as shown in Figure 5. The responsivity at 338 nm is as high as 10.4 A/W biased at 2 V, corresponding to a photocurrent gain of around 40 (Figure 6a). The CaF_2_ layer behaves as an excellent insulator for the InGaN-based MSM photodetectors in dark condition, while it allows the electron injection through the metal/semiconductor interface under UV illumination, contributing to the photocurrent gain without sacrificing the response time. Figure 6b reveals the scheme of the time response at the bias voltage of 5 V. As can be seen, the electrical current drops by more than two orders of magnitude within 0.3 s (limitation of the measurement system) once the UV light is mechanically turned off. A rising response time of 747 μs under the bias voltage of 0.1 V was obtained, which illustrated a little PPC effect.

#### 2.1.3. III-Nitride Photocathodes

Another candidate in the photodetection field is the photocathode. Different from the above introduced photodiodes which detect photocurrent caused by light irradiation, photocathodes detect the photoemission from a material, and allow a seamless image of the distribution of photoemission directly.

**Figure 7.**
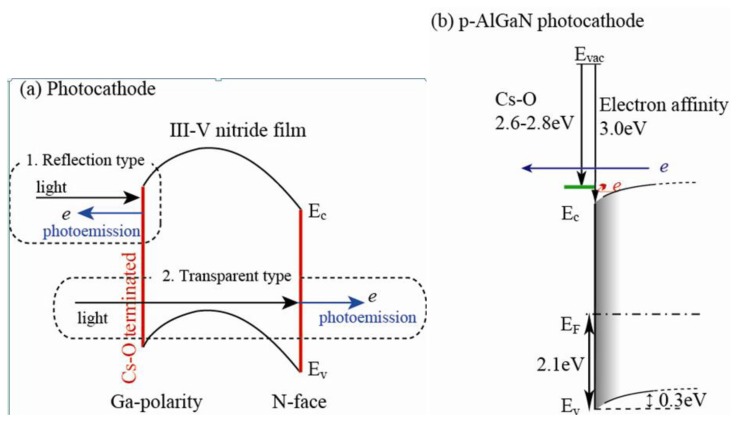
(**a**) Schematic illustration of band diagram to explain reflection and transparent type photocathode device in case of III-V nitride semiconductor. (**b**) Band diagram of p-AlGaNfrom the band-bending value and position of VBM evaluated by hard X-ray photoemission spectroscopy at Spring-8.

In this section, we introduce the potential of photocathode based on III-Nitride films, which has a real benefit of tunable band-gap in term of solar blindness and chemical stability. Conventionally, semiconductors with *p*-type conduction are used for photocathode devices. Downward band bending must be formed at the surface of *p*-type semiconductoras show in Figure 7a, and negative electron affinity (NEA) can be achieved by the application of adatoms onto the material surfaces due to strong dipole moments, e.g., Cs and oxygen [65,66]. There are two types of reflection- and transparence-photocathodes. Photon is emitted in the opposite direction of incident light for the former. The light penetrates through semiconductor active layer, and photon is emitted from its back surface for the latter. Seamless imaging device can be available for the transparent-type photocathode.

We have applied Al_x_Ga_1-x_N films to UV and deep UV photocathodes by controlling the AlN mole fraction which determines the threshold wavelength. Al_x_Ga_1-x_N doped slightly with Mg dopant (∼10^18^ cm^−3^) were grown on Si (111) substrates by a horizontal MOCVD system. AlN/GaN (16 nm/84 nm) multilayers were inserted between Al_x_Ga_1-x_N and the Si substrates in order to avoid cracking the active layer due to large difference of thermal expansion coefficient. The sample was mounted on a cathode electrode in a cylindrical quartz tube. An anode electrode with a 5 mm ϕ hole at the center was placed on the opposite side of the cathode, with a 5 mm gap. The sample was heated in the quartz tube using a tungsten filament to clean its surface and to activate Mg dopant. The surface was treated by the sequential adsorption of Cs and oxygen to enhance the photoemission. Monochromatic light from deuterium and tungsten lamps was irradiated onto the sample through the hole in the anode, and the EQE of the photocathode was analyzed by detecting the photoemission current.

**Figure 8.**
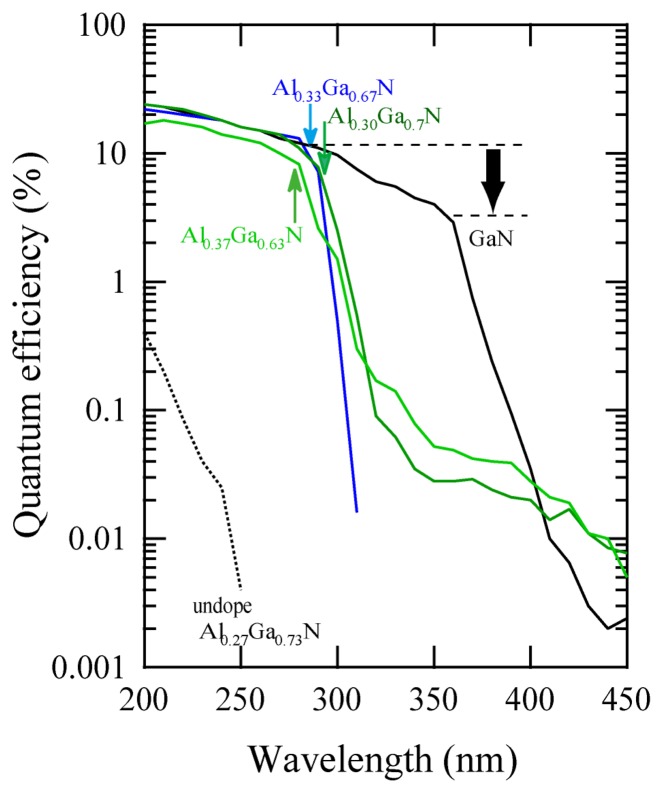
Wavelength dependence of quantum efficiency for photocathodes of Mg-doped Al_x_Ga_1-x_N films on Si (111) substrates (reprinted from Reference [67]).

Figure 8 shows the wavelength-dependence of QE for photocathode devices using Al_x_Ga_1-x_N active layers. The EQE of an undoped AlGaN photocathode was quite low (0.4%) at 200 nm, and dropped dramatically at longer wavelengths due to the potential barrier of *n*-type semiconductor caused by upward band bending at the surface. On the other hand, Mg-doped samples show approximately 20% EQE at 200 nm, and their QE values decreased rapidly for light-irradiation longer than the threshold wavelength. The threshold wavelengths shifted to shorter wavelength with increasing AlN mole-fraction. For an Al_0.33_Ga_0.67_N sample, the value of QE was as high as 20% at 200 nm and 15% at 280 nm, and the on-off ratio between 280 nm and longer wavelengths was more than four orders of magnitude, which was high enough for solar blind sensitivity. The valence band maxima (VBM) at the surface for *p*-Al_0.37_Ga_0.63_N were evaluated at 2.1 eV below the Fermi level, and the surface was bended downward by 0.3 eV [67]. The surface band profile must be formed as illustrated in Figure 7b, taken the values of both electron affinity of 3.0 eV for Al_0.37_Ga_0.63_N, and the dipole momentof ∼2.8eV induced by Cs-O adsorption into account [65–70]. Smaller barrier is still formed instead of the Cs-O dipole moment. This is the reason why the EQE decreases for the photon energy close to the band gap. Surface treatments on the active layer are well known to be crucial for the case of GaAs photocathode. Further optimization of Al_x_Ga_1-x_N surface is necessary to enhance the Cs-O dipole effect, which will result in keeping the QE higher for all ranges of photo energy up to band gap.

**Figure 9.**
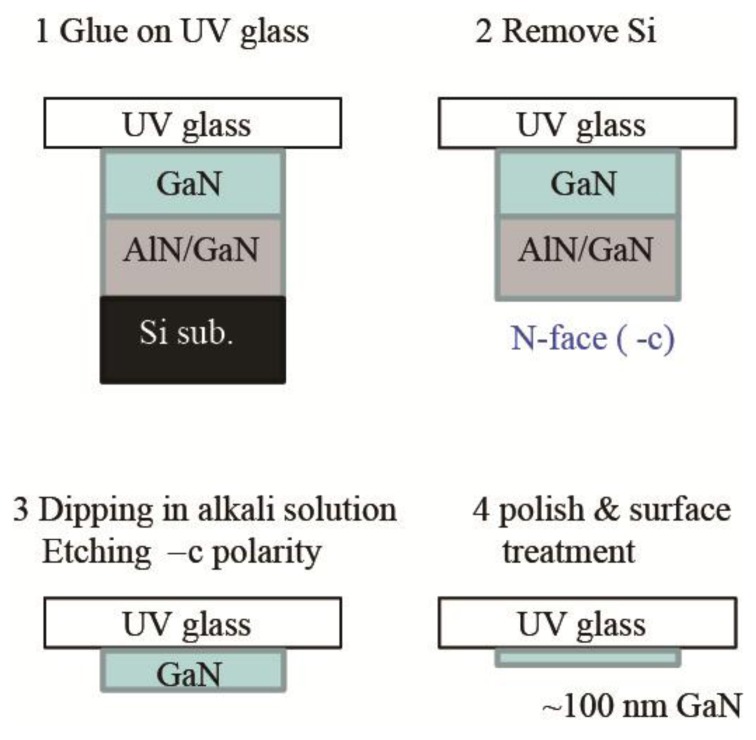
Fabrication process of transparent photocathode based on GaN film grown on Si substrate.

Figure 9 shows the process of transparent photocathode. III-V nitride film grown on Si substrate is glued on UV transparent plate (e.g., SiO_2_), and then Si substrate is removed. Since the nitride film grows along Ga-face (+c) polarity, N-face (−c) polarity surface is exposed on the SiO_2_. III-V nitride film with −c polarity is easily etched in alkali solution [71]. III-V nitride film is thinned down to ∼0.1 μm for sufficient light to reach the surface of the active layer without being absorbed. Since −c polar surface is negatively charged, the surface band bending must be enhanced as shown in Figure 7a, which is another advantage of III-V nitride. As shown in Figure 8, the EQE for GaN photocathode was drastically degraded down to 3%. However, after optimization of the surface treatment, the transparent photocathode with high QE (∼40%) at the band gap energy has been developed, which is available as an imaging intensifier.

### 2.2. SiC-Based Photodetectors

Silicon carbide (SiC), a material long known to have potential for high-temperature, high-power, high-frequency, and radiation hardened applications, has emerged as the most mature wide bandgap (2.0 eV ≤ *E_g_* ≤ 7.0 eV) semiconductor since the release of commercial 6H-SiC bulk substrates in 1991 and 4H-SiC substrates in 1994. The *p*-type and *n*-type SiC can be realized by doping with Al and N, respectively. Commercial instruments, such as SiC-based flame detectors working in the UV-C range, have been developed. However, to select the photodetectors to the appropriate spectral range, the insertion of the high-pass optical filters is necessary.

Since there is little Fermi-level pinning, the Schottky barrier height on SiC depends mainly on the work function of the metal. 6H-SiC Schottky photodiodes were based on *n*-type or *p*-type SiC, working in the 200–400 nm spectral range [72,73]. Anikin *et al.* reported the high-quality Schottky junction on *n*-type SiC with the Schottky barrier height of 1.4–1.63 eV by using Au [74]. The device showed a very low leakage current in the order of 100 pA for a reverse bias voltage of −100 to −170 V, and a high responsivity of 150 mA/W at 215 nm.

Many results on MSM detectors based on 4H-SiC have been reported using semitransparent interdigitated electrodes, such as Cr/Au, Ni/ITO or self-aligned Ni_2_Si [75–77]. By designing the appropriate widths of spacing and opaque interdigitated fingers, the 4H-SiC-based MSM photodetector showed a very low dark current of 0.25 pA at 5 V bias voltage, a typical responsivity of 0.103 A/W at 20 V, and a peak response wavelength at 290 nm [78]. The fabricated devices held a high deep UV to visible rejection ratio of more than 10^3^.

The *p-i-n* junction photodetectors fabricated with SiC material illustrate a low-noise and high-speed response at low reverse bias due to a low terminal capacitance and a large shunt resistance. They can work insensibly to visible/IR backgrounds without visible-blind interference filter. The 6H-SiC UV *p-i-n* photodiodes had already been fabricated and commercially available [79]. The n^+^ layer is always heavily doped to ∼10^19^ cm^−3^. The SiC UV photodiodes showed an extremely low reverse current, and typical peak responsivity of 150–175 mA/W range at 270 nm, corresponding to a 70%–85% quantum efficiency. With an optimized structure design, the 4H-SiC *p-n* junction UV-photodetector achieved a spectral responsivity of 0.03 A/W at 280 nm, and the photocurrent was found to be four orders of magnitude larger than the dark current [80].

Avalanche photodiodes (APDs) present the advantages of high speed, high sensitivity, and large optical gain. 4H-SiC material has a large ionization coefficient ratio ∼10 between holes and electrons, which makes it a good candidate for low noise and high gain. 4H-SiC APDs based on *p-n* or *p-i-n* structures have been reported. Liu *et al*. reported a 4H-SiC *p-i-n* APD with dark current density of 63 nA/cm^2^, and quantum efficiency of ∼40% [81]. By improving the design with the recessed-window structure, and optimized antireflection coating layer, the dark current was reduced to 90 pA with a photocurrent gain of 1,000 [82]. A peak responsivity of 136 mA/W at 262 nm was achieved, with an EQE of 60%, avalanche gains of over 10^6^, an excess noise factor characterized by *k* value of ∼0.1, and a spatially uniform response. The performance was further improved by the separate absorption and multiplication structure [83]. A gain higher than 1.8 × 10^4^ was achieved at 90% breakdown voltage of ∼55 V. At −42 V, the peak responsivity increased to 0.203 A/W at 270 nm, corresponding to a maximum EQE of ∼93%.

### 2.3. Diamond Photodetectors

Diamond (with a wide bandgap of 5.5 eV), with extreme properties such as a high thermal conductivity, high carrier saturation velocity and mobility, offers the highest figure-or-merit for high-performance deep UV detectors meeting the requirements of *5S* [4]. However, it is difficult to utilize polycrystalline diamond to meet the *5S* requirements due to the existence of grain boundaries and non-diamond impurities [84]. Although sulfur-doped diamond UV sensors have shown high sensitivity, high speed, and relative blindness to visible radiation, these defects greatly affect the improvement of the photoresponse [85–87]. On the other hand, single crystal diamond is highly desirable to eventually satisfy the *5S* requirements. The fabrication of diamond devices requires the development of reliable and reproducible doping procedures. *P*-type conductive diamond can be achieved by using boron with an activation energy of 0.37 eV [88]. However, *n*-type doping is difficult because of the close packing and rigidity of the diamond lattice. Efficient incorporation of P has only been achieved on (111) oriented surfaces [89,90]. The reported diamond-based photodetectors including our work focused on the interdigitated-finger MSM photoconductor, MSM photodiode, and Schottky photodiode. These devices were mostly fabricated on the lightly boron-doped diamond thin film, which was grown on the type-Ib diamond substrates by microwave plasma-enhanced chemical vapor deposition (MPCVD) system. The as-grown diamond surface was basically hydrogen-terminated and an oxidation treatment of the epilayers using boiled acid solution of H_2_SO_4_, and HNO_3_ led to oxygen-terminated surface.

**Figure 10.**
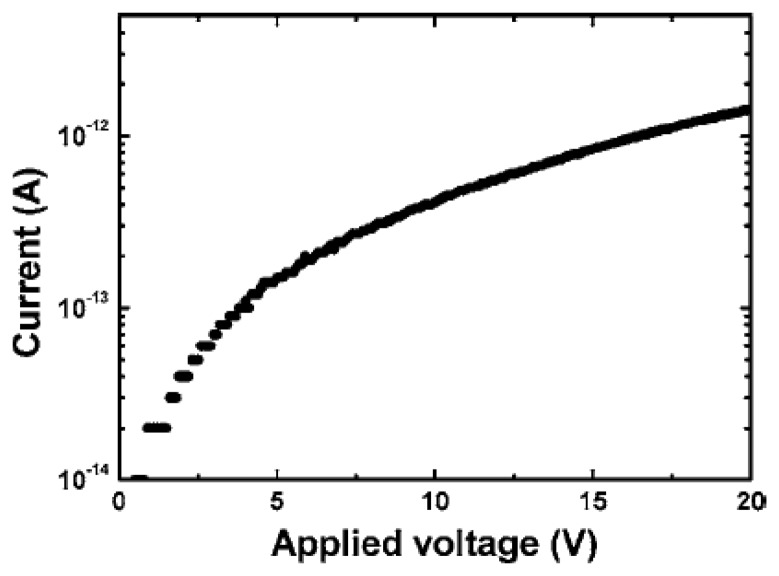
Dark *I-V* characteristics of the MSM diamond photoconductor (reprinted from Reference [91]).

Normally the photoconductor is best suited for high photosensitivity, while a strong PPC was appeared. Due to the high resistivity of the epilayer, the photoconductor fabricated on an oxidized epilayer had a low dark current (1 pA) at 20 V. The dark *I-V* curve disclosed a space-charge-limited-current behavior, consistent with Ohmic contacts to a highly resistive material, as shown in Figure 10 [92].

**Figure 11.**
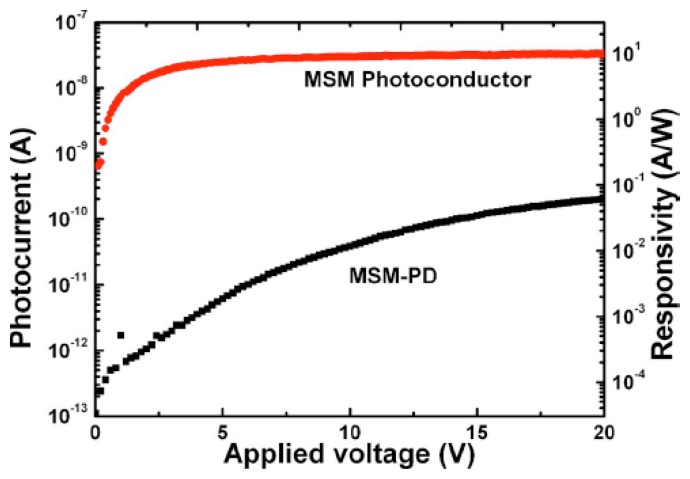
*I-V* characteristics of the photoconductor in comparison with a typical MSM photodetector during 220 nm light illumination (reprinted from Reference [91]).

**Figure 12.**
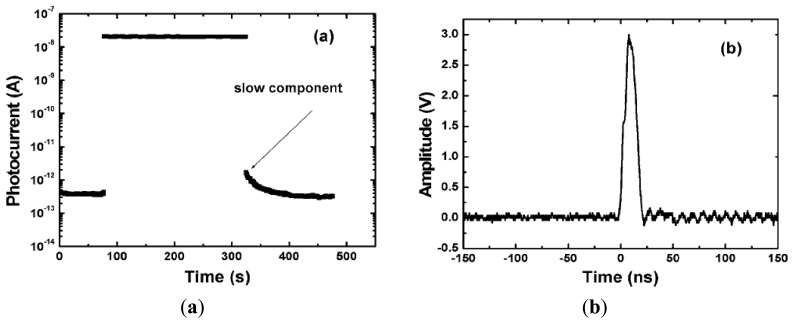
(**a**) Time response of the photoconductor upon 220 nm light illumination measured by a mechanical chopping method and (**b**) time-resolved photoresponse upon a 193 nm excimer laser recorded by an oscilloscope with a 50 Ω impedance (reprinted from Reference [91]).

Figure 11 presents the *I-V* characteristic of the MSM photoconductor upon 220 nm light illumination in comparison with a typical as fabricated MSM photodiode. A responsivity of 6 A/W at 220 nm was obtained for the photoconductor at a bias of 3 V, indicating a gain of 33, if assuming the external quantum efficiency unity. The diamond photoconductor also illustrated a fast response speed, as shown in Figure 12.

The predominant response time at the rising and falling stages was beyond the measurement system time constant of 0.3 s. The possible origins were boron- related impurities or surface traps. The weak PPC showed that the density of the trap states was rather low. The response to a 193 nm laser was also measured, with the full width at half maximum (FWHM) of the photoresponse around 10 ns. The MSM photodiode has the advantages of fabrication simplicity, high bandwidth capability, and suitability for integration with field effect transistor. Al or WC were used as the back-to-back contacts [92,93]. The state-of-the-art diamond MSM photodetectors including ours had been fabricated with the electrode spacing in the scale of several microns [91]. To achieve low-voltage operation, one strategy is to reduce the electrode spacing.

**Figure 13.**
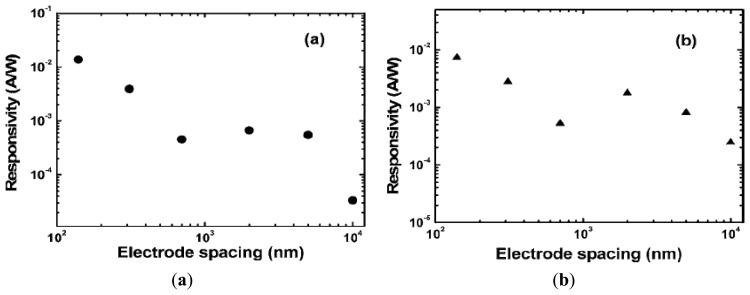
Dependence of the responsivity at 220 nm light on the electrode spacing (**a**) at a fixed voltage of 1 V and (**b**) at an electric field of 2 × 10^4^ V/cm (reprinted from Reference [93]).

We fabricated MSM photodetectors with electrode spacing from 0.14 to 10 μm on a homoepitaxial diamond thin film grown on a Ib-type diamond substrate [93]. A dramatic increase of the deep ultraviolet responsivity is observed when the electrode spacing is scaled down. The reduction in the electrode spacing enables the full depletion of the spacing at low biases, providing a higher responsivity without sacrification of the response speed. Due to the low flatband voltage for the small electrode spacing, the EQE is significantly enhanced from around 0.02% to 8% as the spacing scales from 10 to 0.14 μm at a fixed voltage of 1 V, as shown in Figure 13a. The dramatic increase of the responsivity with the spacing reducing was also observed for a fixed electric field of 2 × 10^4^ V/cm (Figure 13b).

The existing planar Schottky photodiodes for diamond and other semiconductors are usually constructed from an Ohmic contact and a semi-transparent dotted Schottky contact [94–96]. The diodes present low leakage current and high visible rejection, comparable to the best photoconductor on diamond. For the sake of high photosensitivity and high response speed, Schottky photodiodes with interdigital Ohmic and Schottky contacts were created in our group [97].

**Figure 14.**
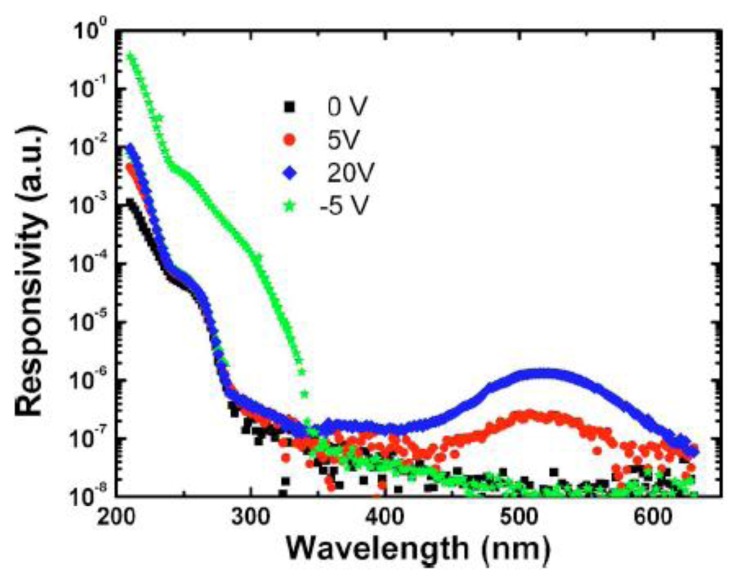
Spectral response of a Schottky detector with a semitransparent dotted Schottky contact at various biases (reprinted from Reference [97]).

It was demonstrated that this device could be operated in both reverse and forward modes for high speed and high responsivity, respectively. It combined the advantages of the MSM photodiode and photoconductor. This device structure enables the operations in both photoconductive mode with large photocurrent gain and depletion mode with fast response speed. Figure 14 shows the spectral response from the devices under both forward and reverse voltages, indicating a good performance as both the photoconductor and photodiodes.

Although there is difficulty in *n*-type doping for single crystalline diamond, there are reports on the diamond *p-i-n* photodiodes [98]. The detectors exhibited a high responsivity of 10∼30 mA/W at 200 nm illumination and demonstrated a visible rejection ratio (200 nm *versus* 500 nm) of six orders of magnitude. It was also demonstrated that these *p-i-n* diamond photodiodes were sensitive in the 200 to 220 nm range, stable under brief irradiation with a good linearity and homogeneity.

### 2.4. One-Dimensional Nanostructured UV Photodetectors

In the last few years, one-dimensional (1D) or quasi-one-dimensional nanostructures have been widely studied and developed as potential candidates for high-performance UV photodetectors. Their small size allows the nanostructures to exhibit novel and significantly modified physical, chemical, and biological properties, which are different from those of materials in the micrometer scale. As a result of the large surface-to-volume ratio and small dimensions, the nanostructured materials are especially sensitive to the light (photoconductivity). Moreover, the possibility to integrate functionality in nanostructures, such as homo- and hetero-junctions, within single nanowires or nanobelts, enables large scale integration. In this section, we will introduce the state-of-the-art photodetectors based on 1D nanostructures working in the UV regions, including ZnO-based, GaN-based, and other metal-oxide-based nanowires, nanobelts, or nanorods.

#### 2.4.1. ZnO-Based Nanostructured Photodetectors

Nanostructured ZnO materials have received broad attention due to their distinguished performance in the photonic and electronic field. ZnO has a wide bandgap of 3.34 eV at room temperature (RT), which makes it very suitable for photodetection working in the UV-A region from 320 to 400 nm. Wurtzite ZnO has a hexagonal structure with lattice parameters *a* = 0.3296 and *c* = 0.5207 nm. The large ionic component of Zn and O, along with the deviation of their equilibrium lattice structure from the ideal wurzite crystal, results in the strong piezoelectric and pyroelectric properties and the consequent use of the ZnO material in mechanical and piezoelectric sensors. Another important characteristics of ZnO is its large exciton binding energy (60 meV), which can ensure efficient excitonic emission at RT. The UV luminescence has been reported in disordered nanoparticles and thin films. Due to its wide bandgaps, ZnO is transparent to visible light, and can be made highly *n*-type conductive by doping [99]. In addition, ZnO nanostructures have unique advantages including high specific surface area, low toxicity, chemical stability, electrochemical activity, and high conductivity [100–103]. Therefore, they are promising material for sensors applications with a high performance.

The synthesis of ZnO started with the thin film growth in the 1960s. Over the last decades, ZnO nanostructures have been of growing interest due to their wide applications. As a result of its versatile functional properties, ZnO exhibits a diverse group of growth morphologies, such as nanocombs, nanorings, nanohelixes/nanosprings, nanobelts, nanowires, nanotubes, nanotips, nanoflows, nanosheet, nanoporous, nanocages, *etc*., whose electrical and optical characteristics are quite different. In the recent years, the research on 1D ZnO-based nanostructures has rapidly expand. As pointed out by Wang *et al*. and Fang *et al*. in the latest reviews, 1D ZnO-based nanostructure has been one of the few dominant nanomaterials in nanotechnology together with carbon nanotubes and silicon nanowires [100,104].

**Figure 15.**
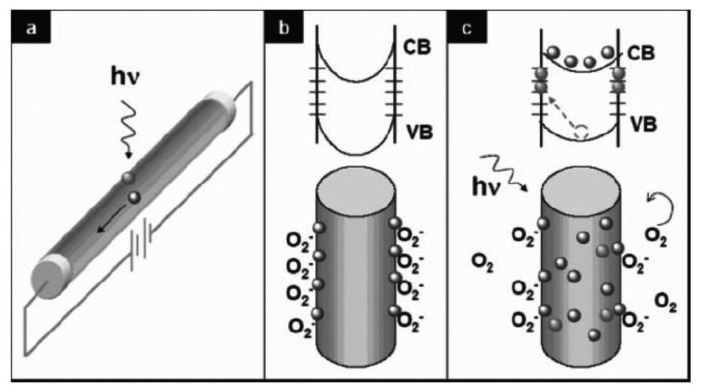
Photocurrent transport mechanism in a nanowire (reprinted from Reference [105]).

Because of the high surface-to-volume ratio, and superior properties, ZnO has been widely developed for nanometer-scale visible-blind UV-light detection with a high sensitivity and selectivity. The photoconduction mechanism in a nanowire is shown in Figure 15[105]. When the nanowire is upon the UV light illumination, the electron-hole pairs are generated. Holes are trapped at the surface due to the surface trap states (oxygen molecules which are absorbed at the surface). Under the applied voltages, the unpaired electrons are collected at the anode, increasing the conductivity. The first individual ZnO-based nanowire photodetectors were reported by Yang *et al*. in 2002 [106]. The conductivity of the ZnO nanowires was extremely sensitive to UV light exposure (365 nm), with a typically four to six orders of magnitude decrease in the resistivity. The device showed a high response speed with the rise and decay time below the detection limit (1 s) in spite of the high responsivity. The photoresponse was strongly dependent on the ambient gas condition, being slow in vacuum and inert gases and fast in air, indicating the role of the oxygen for the photocurrent gain.

Great progress has been achieved since the year 2002 to improve the sensitivity to UV light based on ZnO-based nanostructures. For example, by polystyrene surface functionalization, the UV photoresponse of ZnO nanowire has been enhanced by three orders of magnitude as compared to that without surface coating [107]. It is found that the dark conductance of the device decreased by three orders of magnitude after surface coating while the light conductance shows little variation. Lao *et al.* demonstrated that the UV response of a ZnO nanobelt-based detector had been enhanced by nearly five orders of magnitude after functionalizing its surface with a polymer with a high UV absorption ability [108]. This giant enhancement in photoconductance was attributed to the electron-hole generation process as assisted by the energy levels states introduced by the polymer lying in the corresponding band gap of ZnO. These studies suggest that, highly sensitive UV detectors with a large range of detection wavelength can be fabricated using ZnO nanostructures by polymer surface-functionalization. Aluminium-doped ZnO nanorods arrays were also developed for UV photodetection [109]. The nanorod arrays were prepared using a sonicated sol-gel immersion and annealed at 500 °C under air and oxygen ambient. The annealing process induced the formation of nanoholes on the nanorod surface, which increased the nanorod surface area. It was shown that annealing in the air produced the large nanoholes compared with those annealed in the oxygen ambient. Annealing reduced the rise and decay time constants of the UV sensor. A high responsivity of 1.55 A/W for UV light under 10 V was achieved for the sample annealed in an oxygen atmosphere, which is mainly due to defect reduction and improvement in stoichiometric properties. Another kind of ZnO nanowire-based UV detector was achieved through fabrication a Ag/ZnO heterostructure by a photoreduction reaction to form the Schottky contact by the formation of AgO_x_, which led to a significant enhancement in the performance [110]. An efficient and low-working-temperature method was proposed for eliminating the persistent photoconductivity in ZnO nanostructure-based photodetectors, and the approach could be widely applied to other photodetectors employing the Schottky barrier structures.

#### 2.4.2. GaN-Based Nanostructured Photodetectors

Like their thin film structures, III-Nitride based nanostructures whose cutoff wavelength is variable by using InGaN or AlGaN alloys have also been attracting more attention for UV photodetection. The photoconduction of the single GaN nanowires has been investigated by many groups [111]. It has been found that the sensitivity is dependent on the column diameter. Networked GaN nanowires showed strong sensitivity to the ambient conditions.

**Figure 16.**
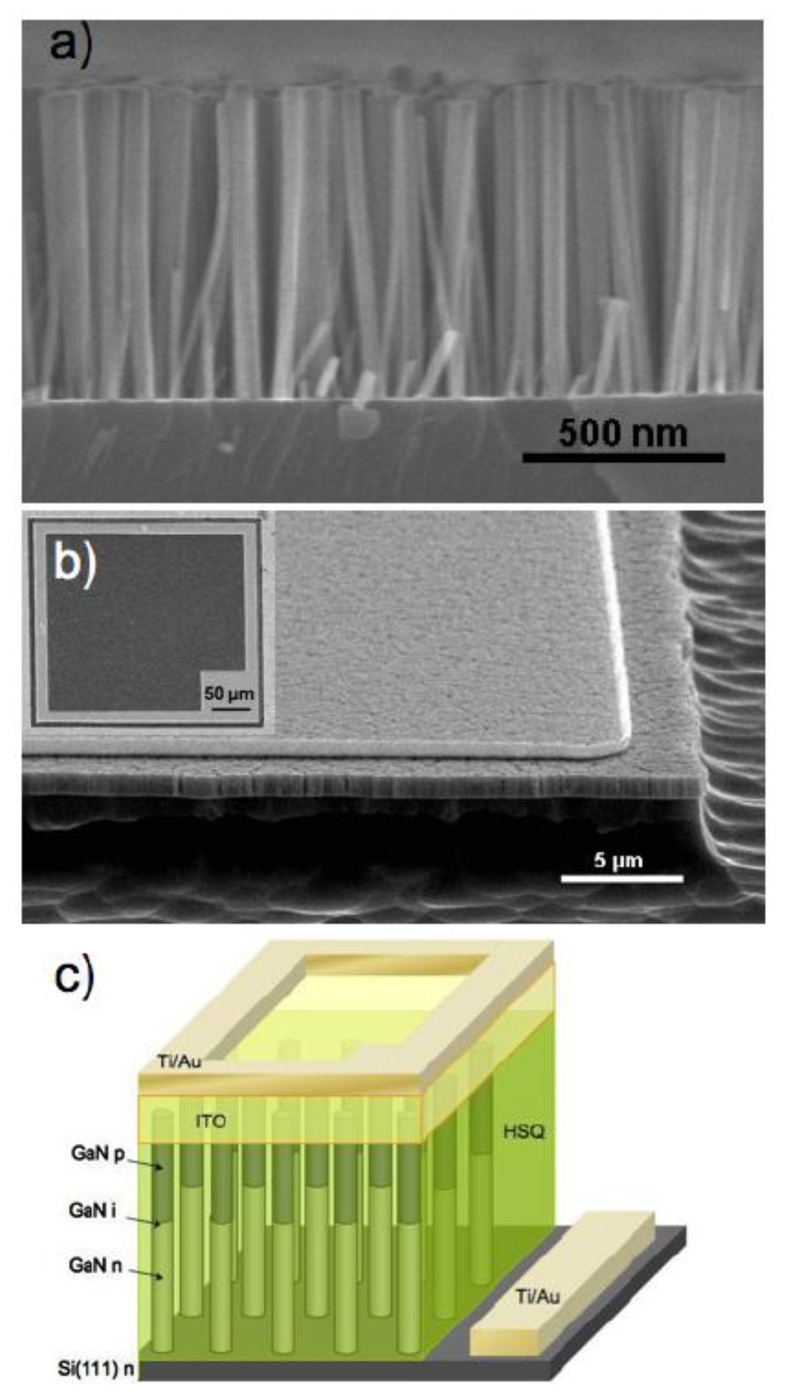
(**a**) Cross-sectional SEM image of as-grown nanowires; (**b**) 45°-tilted SEM image of a mesa photodetector; (**c**) Schematic representation of a mesa photodetector (reprinted from Reference [113]).

GaN nanostructured *p-n* junctions have been achieved in nanorods and nanowires. Son *et al*. reported single GaN nanorod *p-n* junctions which were grown by using the plasma-assisted molecular beam epitaxy (MBE) [112]. In order to form the *p-n* junction at the middle of the nanorod, the dopant was changed from Si (*n*-type) to Mg (*p*-type) during the growth procedure. The rectifying behavior was observed for the single GaN nanorod *p-n* junction under the dark condition. When the UV-light at 365 nm was applied, the photocurrent increased. The photoresponse on/off ratio was estimated to be about 14 at −0.03 V. The visible-blind photodetector based on *p-i-n* junction GaN nanowire ensembles were achieved by Bugallo *et al*. [113]. The nanowires were grown by plasma-assisted MBE on an *n*-type Si substrate. Figure 16 shows a cross-sectional SEM image of the as-grown nanowires, and the schematic representation of a photodetector device. The detector presented a high peak responsivity of 0.47 A/W at −1V, which was higher than that of the thin film GaN *p-i-n* photodetectors. The spectral response of the detector was restricted to the UV range with a UV-to-visible rejection ratio of more than 10^2^. The photodetector response was linear over two decades of incident power density up to 6 × 10^−4^Wcm^−2^.

Rigutti *et al*. demonstrated a single-nanowire photodetector which relied on carrier generation in GaN/AlN quantum discs [114]. The doped extremities of the nanowires provided an electrical access allowing probing of the carrier photogeneration in the quantum discs region. Two heterostructured nanowire samples containing quantum discs of different thickness were analyzed and compared to a reference binary *n-i-n* GaN nanowire sample. The responsivity of a single wire disc detector was measured to be as high as 2000 A/W at 300 nm illumination at RT. Photocurrent spectroscopy allowed identification of the spectral contribution related to carriers generated within large discs, which lies below the GaN band gap due to the quantum confined Stark effect.

#### 2.4.3. Other Metal-Oxide Nanostructured Photodetector

Due to their special shapes, compositions, and chemical and physical properties nanostructured metal-oxides are the focus of current research interest in nanotechnology since they are the most common minerals on the Earth. They have potential applications widely in many fields, such as transparent electronics, ceramics, catalysis, sensors, transducers, or electro-chromic devices. There is detailed review on the 1D metal-oxide nanostructures and their applications [9]. In this article, we focus on their applications in the UV photodetection field considering on the wide-bandgap 1D nanostructures, including ZnO (hexagonal, 3.37 eV), Ga_2_O_3_ (Monoclinic, 4.2–4.9 eV), SnO_2_ (Tetragonal, 3.6 eV), and In_2_O_3_ (Cubic, 3.6 eV). The ZnO-based nanostructured photodetectors have been introduced in Section 2.4.1. In this section, we will focus on other metal-oxide nanostructures such as Ga_2_O_3_, SnO_2_, In_2_O_3_, ZnGeO, and In_2_Ge_2_O_7_ nanostructured UV photodetectors.

Monoclinic gallium oxide (*β*-Ga_2_O_3_) semiconductor, with the wide bandgap of ∼4.9 eV in the deep UV region, is one of the most promising candidates for high-performance UV photodetectors. Individual *β*-Ga_2_O_3_ nanostructures such as nanobelts and nanowires, have been demonstrated by several reports to be potential solar-blind UV photodetectors [115–120]. The first individual *β*-Ga_2_O_3_ was reported in 2006 by Feng *et al*. The detectors showed encouraging advantages to 254 nm light illumination. The dark current was on the order of pA, which was one of the best properties of *β*-Ga_2_O_3_ nanostructures. The conductance of the nanowire in deep UV region was about three orders of magnitude higher. The upper limits of the response and recovery time were 0.22 and 0.09 s, respectively.

**Figure 17.**
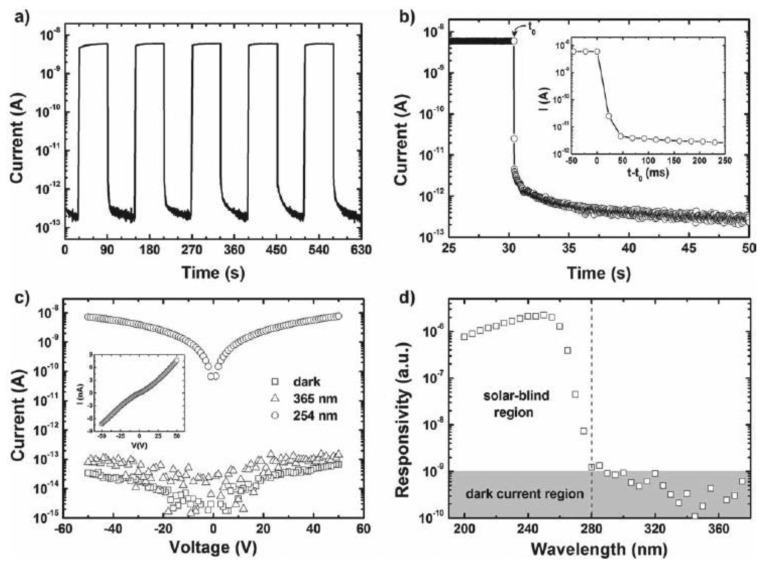
Photoresponse properties of the bridged *β*-Ga_2_O_3_ nanowires. (**a**) Time-dependent photoresponse measured in the air under 254 nm illumination; (**b**) Photocurrent decay process of the device; (**c**) I-V characteristics of the device in the dark, under 365 nm and 254 nm light illumination; (**d**) Spectral response of the bridged *β*-Ga_2_O_3_ nanowires revealing that the device is blind to solar light (reprinted from Reference [117]).

Li *et al*. reported the assembly of *β*-Ga_2_O_3_ nanowires into high-performance solar-blind photodetectors by use of an efficient bridging method [117]. The photoresponse properties of the bridged *β*-Ga_2_O_3_ nanowires are shown in Figure 17. The dark current was only ∼0.2 pA under a bias of 50 V, which was favorable for practical sensing devices. Upon 254 nm illumination, the current instantaneously increased by more than four orders of magnitude. The high photoconductivity of the *β*-Ga_2_O_3_ nanowires might be attributed to the surface states arose from the amorphous surface layer, which served as trapping centers for the photogenerated holes, caused charge separation, and prolonged the lifetime of the photogenerated electrons.

The PPC effect was not observed in current *β*-Ga_2_O_3_ nanowire photodetectors. The photoresponse spectrum showed a good selectivity of the devices, in which, the maximum responsivity was found to be ∼250 nm. A large 250-to-280 nm rejection ratio of more than a 1,000 was obtained, which was much higher than other reports. The photoresponse mechanism of the *β*-Ga_2_O_3_ nanowires was discussed in detail in [116].

Niobium pentoxide (Nb_2_O_5_), which is one of the most important transition metal oxides, is an ideal candidate for visible-blind UV sensors (with a bandgap of ∼3.4 eV). Fang *et al.* developed a facile topochemical process to synthesize quasi-alighed Nb_2_O_5_ nanobelts arrays with a controlled morphology and structure, and fabricated photodetector from an individual nanobelt [121]. The photodetector showed a high UV-A-light sensitivity, high external quantum efficiency of 6070%, and excellent photocurrent stability of more than 2,500 s. Photoresponse of isolated Nb_2_O_5_ nanowires were also studied by using global and localized focused laser beam irradiation by Tamang *et al*. [122]. A fast and prominent photoresponse was found towards visible and infrared laser irradiation.

**Figure 18.**
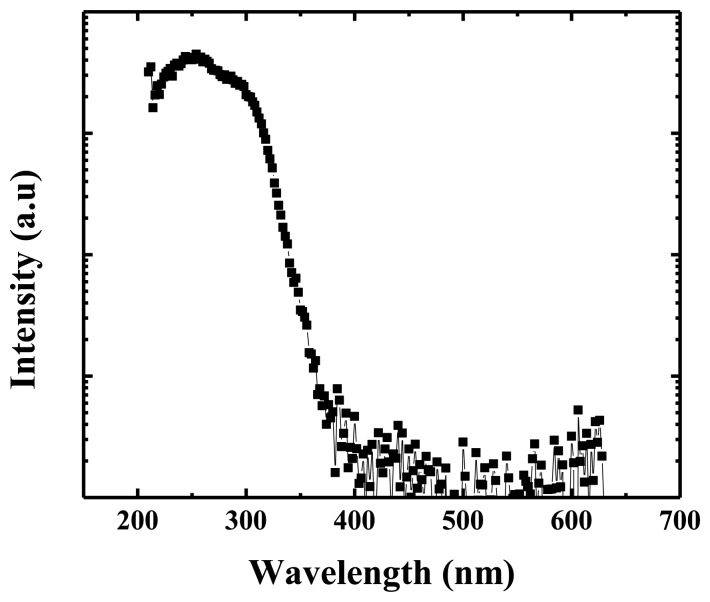
Typical photoresponse spectrum of SnO_2_ nanowire.

SnO_2_, another important *n*-type metal-oxide semiconductor, has been developed widely in the applications of photodetectors, chemical sensors, transparent conducting electrodes, *etc*. In 2009, Wu *et al*. reported photodetectors made from SnO_2_ nanowires [123]. The photoelectric current of the SnO_2_ nanowires exhibited a rapid photo-response as a UV lamp was switched on and off. Hu *et al*. reported on the ultrahigh external quantum efficiency (EQE) from thin SnO_2_ nanowire UV photodetectors [124]. Nanowire with a uniform diameter of about 26 nm was synthesized in a horizontal quartz tube furnace at 910 °C. The resulting photodetector exhibited excellent light selectivity and stability, and an ultrahigh EQE value of 1.32 × 10^7^. A high-quality SnO_2_ hollow-sphere based flexible photodetector was reported by Tian *et al*. [125]. The photodetector showed high sensitivity, super stability, and was also able to bear significant external mechanical forces. Figure 18 shows the typical photoresponse spectrum for a single SnO_2_ nanowire.

In_2_O_3_ is a wide-bandgap semiconductor with a direct bandgap of ∼3.6 eV, and indirect bandgap of ∼2.5 eV. The bulk In_2_O_3_ has been demonstrated to work as an ultrasensitive detector due to the surface interaction. 1D In_2_O_3_ nanostructures are expected to offer enhanced sensitivity and an improved response time due to their increased surface-to-volume ratios. In_2_O_3_ nanowires were synthesized using a laser ablation technique via the vapor-liquid-solid mechanism [126–128]. Zhang *et al*. demonstrated the UV photodetection properties of In_2_O_3_ nanowires. Devices based on individual In_2_O_3_ nanowires showed a substantial increase in conductance of up to four orders of magnitude upon UV light illumination. UV light with a wavelength of 365 nm brought a current of ∼33 nA, and a 290 nA photocurrent occurred when the device was exposure to 254 nm. Such devices also exhibited short response times and significant shifts in the threshold gate voltage. The authors have also demonstrated that the UV light can be used as a “gas cleaner” for In_2_O_3_ nanowire chemical sensors, leading to a recovery time as short as 80 s. Recently, Shao *et al*. reported on the high photoresponsivity in the near UV range for a photodetector fabricated from polyvinyl-alcohol (PVA)-coated In_2_O_3_ nanoparticles [129]. The photodetector exhibited either a low-pass or bandpass spectral response, depending on the illumination directions.

Ternary metal-oxide alloys are another candidate for nanostructured photodetectors. In_2_Ge_2_O_7_ nanobelts showed the ultrahigh performance for the solar-blind photodetectors. Li *et al*. fabricated high-quality single crystalline In_2_Ge_2_O_7_ nanobelts. The individual nanobelt photodetector displayed a high responsivity (3.9 × 10^5^ A/W), high EQE (2.0 × 10^8^%), excellent stability and reproducibility [130]. Individual Zn_2_GeO_4_ nanowire photodetector displayed an extremely low dark current (<0.1 pA), a responsivity of 38.3 A/W (corresponding to a photocurrent gain ∼200), a high deep UV-to-visible ratio up to 104, and a relatively fast response speed [131]. Feng *et al*. reported on high-performance ZnGa_2_O_4_ nanowires photodetectors working in the deep UV region [132]. The devices showed fast response speed and enhanced photocurrent when the vacuum level was increased. It was found that surface-related processes, especially oxygen chemisorption, had significant effects on the photoelectric properties of the nanostructures.

### 2.5. Hybrid Photodetectors

In recent years, to achieve multiple or wide band absorption, or to realize the better spectrum selectivity independently, hybrid photodetectors are attracting more and more attention. Multicolor optical sensing with high sensitivity at designed wavelengths can be applied in a variety of applications, such as imaging, surveillance, optical communication, remote control, and target identification.

Since the absorption of organic materials can be easily tuned by tailoring the chemical structure, UV sensors based on those materials seems to possess more flexibility in realizing spectra selective responses. Li *et al*. demonstrated a low cost UV sensor based on polymer/ZnO nanorods, and the device showed a narrow-band response in the region of 300–360 nm [133]. The hybrid UV detector composed of TiO_2_ nanorods and polyfluorene showed obvious UV photoconductive effect, and the response was fast to the switching on and off of UV light illumination, which was repeated at least 50 times [134].

Although ZnO-based semiconductors have been recognized as very promising photonic materials in the UV region, its *p*-type doping remains a challenge. Hybrid *p-n* junction photodetectors based on *p*-GaN/*n*-ZnO or *p*-type polymer/*n*-ZnO are another research topic. Li *et al*. proposed a nanostructured near UV photodetector based on ZnO nanorod/polyfluorene hybrid devices, which demonstrated a high responsivity of 0.18 A/W at 300 nm [135]. The *n*-ZnO films had been deposited onto *p*-GaN to form a *p-n* junction, and the *I-V* curves showed obvious rectifying behaviors [136]. Under back-illumination conditions, the GaN layer on one hand acted as a *p*-type counterpart for the *n*-ZnO layer, on the other hand as a “filter” that was transparent to the illumination light with wavelengths longer than 360 nm, therefore, the photodetector shows a narrow band-pass response of only 17 nm in width.

A *β*-Ga_2_O_3_/GaN Schottky-Barrier photodetector was fabricated by furnace oxidation with oxygen gas at 1,000 °C for 10 min [137]. It was found that the depletion depth of the device could be controlled and switch the operation mode between solar-blind and visible-blind by changing the applied voltage. A broadband absorption spectrum from IR to UV regions was achieved by using solution-processible PbSe-TiO_2_-Graphene hybrids [138].

On the other hand, the photodetectors fabricated from the heteroepitaxial junctions based on nanostructures are attracting more and more attention recently. Interesting results have been reported by Fang's group. They synthesized GaP/ZnS coaxial nanocables and used them to fabricate UV-light sensors. Compared with the unitary ZnS nanobelts, the GaP/ZnS coaxial nanocables exhibited improved optoelectronic properties such as high photocurrent and excellent photocurrent stability [139]. Side-to-side single-crystalline ZnS/ZnO biaxial nanobelts were demonstrated to be promising for UV A-light sensors by the same group [140]. The photodetector based on this binary ZnS/ZnO biaxial nanobelt exhibited tunable spectral selectivity and wide-range photoresponse, which was much better than that of the pure ZnS or ZnO nanostructures.

We have demonstrated arbitrary multi-color photodetection by hetero-integrated semiconductor nanostructures [141]. The various semiconductor nanostructures were integrated on the wide-bandgap semiconductor or an insulator substrate. The visible light, UV light, and deep UV light three-band photodetectors were created by integrating ZnO sub-microrods, and CdS nanowires on a diamond layer. Because of the photoresponse of each spectra band is determined by each semiconductor nanostructure or the semiconductor substrate, the detectors can achieve a high spectra selectivity, high sensitivity, high speed, high signal-to-noise ratio, high stability and high simplicity.

### 2.6. High-Temperature Stable Photodetectors

Since the UV signal exists in a large solar radiation background, in some cases such as the flame detection for a hot engine, thermally stable detectors with a high performance are required. The reported UV photodetectors which can withstand high temperatures include SiC and SiC-based detectors, diamond solar-blind photodetectors, III-Nitride detectors, *etc*. On the other hand, the 1D nanostructures are sensitive to the temperature due to their strong surface states resulting from the large surface-to-volume ratio. Up to now, there are no reports on 1D nanostructured photodetectors working at high temperatures. In this section, we summarize the state-of-the-art photodetectors which can work at high temperatures. In particular, in our study on InGaN-based visible-blind photodetectors, we found that by using CaF_2_ as the insulation layer, the photodetector could work at a temperature as high as 523 K. We will also introduce our results on high-temperature and high-detectivity solar-blind photodetectors based on *β*-Ga_2_O_3_ nanowires, in which the photoresponse behavior is dominated by the bulk instead of the surface states.

SiC photodiodes are one of the best candidates for high-temperature applications. The high-temperature operation of a UV sensor had been reported by several groups. The highest operation temperature up to now is 700 °C, which was reported by Toda *et al*. by using N^+^ implantation into 6H-SiC [5]. The photocurrent of the sensor increased with temperature, and at 400 °C and 700 °C, the photocurrent was approximately double and triple that at RT, respectively. The temperature dependence of the photocurrent reveals the characteristics of absorption particular to indirect transition, and also the minority carrier diffusion length. Dark current increased rapidly at temperatures exceeding 450 °C.

Silicon carbon nitride (SiCN), a wide-bandgap semiconductor, has many interesting physical characteristics such as hardness, high thermal stability, oxidation resistance, and corrosion resistance. Photodetectors based on SiCN film have demonstrated good thermal stability working in the visible-blind region. Chang *et al*. reported on the MSM structure on a cubic-crystalline SiCN film deposited on Si substrate with rapid thermal chemical vapor deposition [142]. The optoelectronic performance of the SiCN-MSM detector had been examined by measuring the photo to dark current ratio under different operation temperatures. The current ratio for 254 nm UV light was about 6.5 at RT and 2.3 at 200 °C. With effective doping both in *p*-type and *n*-type, the *n-i-p* anb *n-p* SiCN homojunction UV detectors on Si substrate by using the same method also demonstrated good performances [143]. The photo to dark current ratios of the homojunction under −5 V, with and without irradiation of a 254 nm UV light were 3,180 and 135.65, respectively, at RT and 200 °C.

**Figure 19.**
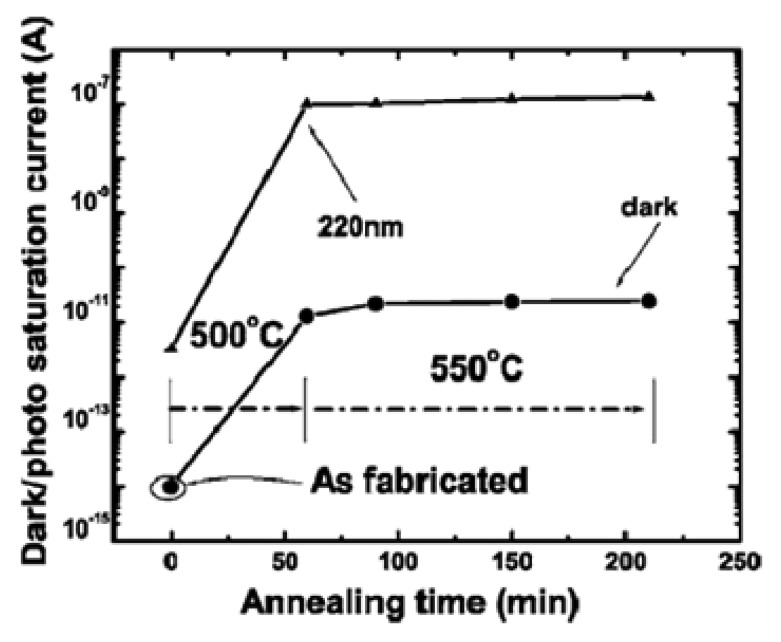
Dependence of the saturated dark current and photocurrent of the diamond photodiode under 220 nm light illumination with an intensity of 26 μW/cm^2^ at a reverse bias of 20 V on the annealing temperatures and times. (Reprinted from Reference [6]).

The superior physical and chemical stability of single-crystalline diamond film favor its use as a high-temperature deep UV photodetector. We have developed a thermally stable photodiode using tungsten carbide (WC) Schottky and Ti/WC ohmic contacts for a boron-doped homoepitaxial *p*-diamond epilayer [6]. The effect of thermal annealing in an argon atmosphere on the electrical and photoresponse properties was investigated (Figure 19). Annealing at temperatures up to 550 °C improved the rectifying *I-V* characteristics, resulting in a dramatic enhancement of deep UV responsivity at 220 nm by a factor of 4,000. A blind ratio as large as 10^5^ between UV and visible light was achieved at a reverse bias as small as 1 V. The development of thermally stable WC-based Schottky and Ohmic contacts provides a route for stable operation of a diamond photodetector at high temperatures.

III-Nitride semiconductors are one of the most promising candidates for high-performance UV detectors due to their direct wide bandgap structure, high thermal stability, radiation hardness, and expected high responsivity. However, due to the high-density defects existing within the hetero-epitaxial structure, III-Nitride-based devices commonly suffer from enhanced leakage currents at elevated temperatures, even for devices using GaN substrates. In order to develop a thermally stable GaN-based photodiode, one should first decrease the reverse leakage current [144]. Feng *et al*. reported AlGaN-based solar-blind MSM photodetectors which worked at a high temperature of more than 150 °C on a high-quality AlN buffer layer [145]. The device exhibited ultra-low dark current in fA range at RT under 20 V bias, and a breakdown voltage higher than 300 V. The EQE at ∼275 nm was around 64% with a UV/visible rejection ratio up to four orders of magnitude. Even at 150 °C, the dark current was still in fA range with a reasonable rejection ratio of more than 8,000, suggesting its potential applications for high-temperature deep UV detection.

**Figure 20.**
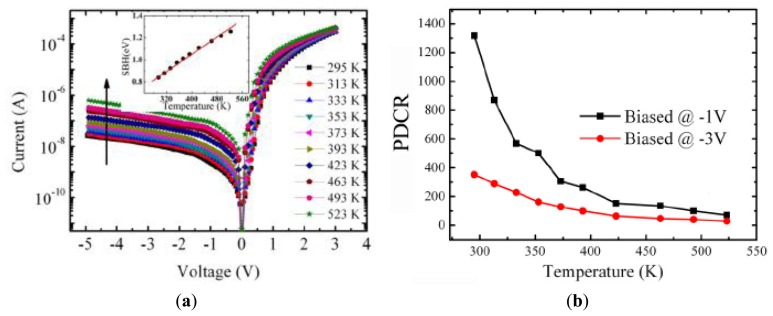
(**a**) Dark I-V characteristics of the MIS photodetector as the temperature was elevated from RT to 523 K; (**b**) The PDCR variation as the measurement temperature at −1 and −3 V. (Reprinted from Reference [8]).

A promising strategy is the adoption of the MIS structure. We demonstrated that by using CaF_2_ as the insulation layer, the InGaN-based visible-blind photodiode showed the good performances at high-temperatures up to 523 K (Figure 20) [8]. The reverse leakage current remained at a low level (10^−7^–10^−8^ A), while the UV responsivity is as high as 5.6 A/W at −3 V under 523 K, without observing the PPC effect. The discrimination ratio between 378 nm UV light and visible light was maintained at more than 10^5^. The photocurrent gain was observed at different temperatures and different voltages, which was interpreted in terms of thermionic-field emission and field-emission tunneling mechanism from RT to 463 K, while thermionic-field emission becomes the dominant mechanism at high temperatures.

**Figure 21.**
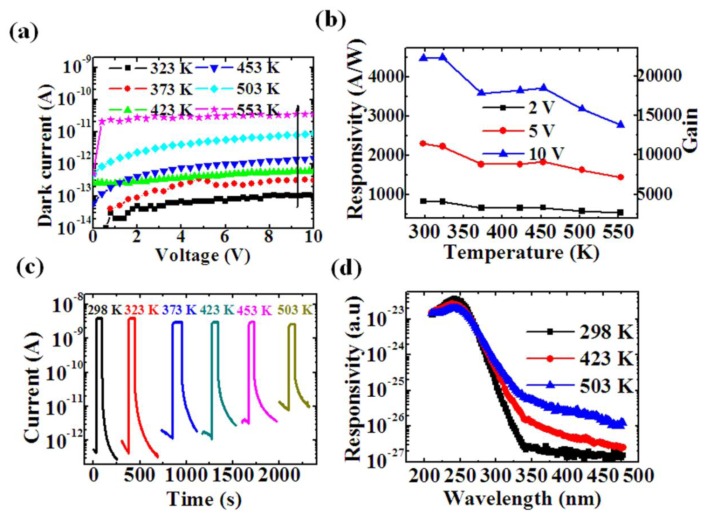
Photoresponse properties of the *β*-Ga2O3 nanowire based photodetectors at various elevated temperatures. (**a**) Dark current-voltage characteristics at various temperatures. (**b**) Responsivity at the 250 nm light illumination dependence on the temperature. (**c**) Time response upon the 250 nm light illumination at various temperatures at 5 V. (**d**) Spectral response measured at 5 V at different temperatures.

Photodetectors fabricated from 1D semiconductors are always dominated by the surface states due to their large surface-to-volume ratio. Therefore, the basic *5S* requirements for practical photodetectors are difficult to satisfy. In our study, we demonstrated the bulk-dominated photoresponse behaviors for the deep UV *β*-Ga_2_O_3_ nanowires photodetector with the high-temperature stability and high detectivity [146]. Ohmic contact to the *β*-Ga_2_O_3_ nanowires was achieved by using a thermally stable tungsten carbide electrode. As a result, the deep UV responsivity at 250 nm shows the highest values of 4,492 A/W at RT, and 3,000 A/W at 553 K. The detectivity is as high as 1.26 × 10^16^ cmHz^1/2^W^−1^ at RT, and still remained 4.1 × 10^14^ cmHz^1/2^W^−1^ at such a high temperature of 553 K. Figure 21 shows the photoresponse properties of the *β*-Ga_2_O_3_ nanowire based photodetectors at various elevated temperatures. The photocurrent dynamics from the *β*-Ga_2_O_3_ nanowire was discussed in terms of the bulk dominated photoresponse behavior. Other wide-bandgap nanostructures based DUV detectors could also be developed for high-temperature application based on this work.

Table 2 summaries the reported thermally stable photodetectors working in the UV region.

## 3. Conclusions and Outlook

We have presented a broad overview of the state-of-the-art progress in the field of UV photodetectors in the visible-blind and solar-blind regions based on wide bandgap semiconductor films and 1D nanostructures, including III-Nitrides, SiC, diamond, and metal-oxides such as ZnO, In_2_O_3_, Ga_2_O_3_, SnO_2_ nanowires or nanorods. The hybrid photodetectors which are aimed at a broad-band absorption are also included. Special sttention is paid to the thermally-stable photodetectors developed from SiC-based materials, GaN-based materials, diamond and bulk-dominated *β-*Ga_2_O_3_ nanowires. This article also lists our recent investigations on UV photodetectors, both semiconductor films (AlGaN-based solar blind photodetctors, InGaN-based visible-blind photodetectors and their thermal stability, diamond deep UV photodetectors) and 1D nanostructures (SnO_2_ nanowires, *β-*Ga_2_O_3_ nanowires and their thermal stability). Needless to say that, this review is unable to list all the exciting works reported in this field due to the space limitations.

It is noted that the photodetectors based on the wide bandgap semiconductors have attained fascinating achievements in the past years. SiC, III-Nitrides, and diamond photodetectors have already been commercialized for applications such as fire sensors, engine control or environmental monitoring. However, they can still not replace silicon detectors in the short term due to the poor reproducibility and reliability of the devices. The development of high-quality substrates for homoepitaxial growth is the key point to improve the reproducibility and reduce costs. 1D nanostructured photodetectors could potentially allow low cost and large scale production on a variety of host substrates. Nevertheless, the reliable control of the diameter, length, orientation, or crystallization is still the big challenge. Furthermore, the device fabrication process is still far from the practical application.

There is still plenty of room for the development of wide bandgap semiconductors and their photodetector applications. Future work should focus on novel concepts both in the materials epitaxy and device fabrications. The combination of the advantages of both thin films and nanostructures will be another interesting topic. It looks promising that many more achievements in UV photodetectors based on wide-bandgap semiconductors will be further attained to meet the demands of various applications, especially at high temperatures and under strong UV irradiation.

## Figures and Tables

**Table 1.** InGaN-based visible-blind photodetector using different insulators.

**Structure**	**Dark Current Reduction**	**UV/ Visible Ratio**	**Responsivity**	**Response Time**	**Thermal Stability**	**Reference**
MSM: SiO_2_**(88 nm)** on GaN/InGaN-MQWs/GaN structure	7 orders of magnitude	10^3^	No.	No.	No.	[60]
MIS: Si_3_N_4_ (**10 nm**) on InGaN/GaN heterojunction	2∼3 order of magnitude	10^3^∼10^4^	0.18 A/W	No.	No.	[61]
MSM: Mg-doped GaN **(40 nm)** on InGaN/GaN-MQWs	6 orders of magnitude	10^3^	0.3∼0.4 A/W @ 4 V	No.	No.	[62,63]
MSM: CaF_2_**(5 nm)** on InGaN thin film	6 orders of magnitude	10^6^	10.4 A/W @ 2 V	∼ms	No.	[64]
MIS: CaF_2_**(8 nm)** on InGaN film	3 orders of magnitude	10^5^	5.6 A/W @ 3 V	<0.3 s	Good more than 500 K	[8]

**Table 2.** Thermally stable photodetectors working in the UV region.

**Structures**	**Dark Current**	**UV/V Ratio at RT**	**Responsivity**	**Response Time**	**Thermal Stability**	**Reference**
SiC: Schottky type	10^−10^ A @ −8 V	No.	58.6 mA/W	No.	973 K	[141]
SiCN: MSM type	10^−5^ A @ −5 V	6.5	No.	No.	473 K	[142]
SiCN: *p-i-n* homojunction	10^−7^ A @ −5 V	3180	0.14 A/W @ 4 V	No.	473 K	[143]

Diamond: Schottkytype	<10^−11^ A @ 32 V	∼10^5^	0.99 A/W @1 V	No.	>800 K	[6]
GaN	No.	No.	No.	∼ms	600 K.	[144]
AlGaN: MSM type	∼fA @ 20 V	10^4^	∼0.14 A/W @ 10 V	No.	323 K.	[145]

InGaN: MIS type using CaF_2_ insulator	1.47 × 10^−10^ A @−5 V	>10^5^	5.6 A/W @ 2 V	∼ms	523 K	[8]

*β*-Ga_2_O_3_ nanowires: MSM type	∼pA	>10^4^	4492 A/W @ 32 V	∼s	553 K	[146]

## References

[1] Lin C.H., Liu C.W. (2010). Metal-insulator-semiconductor photodetectors. Sensors.

[2] Monroy E., Omnes F., Calle F. (2003). Wide-bandgap semiconductor ultraviolet photodetectors. Semicond. Sci. Technol..

[3] Omnes F., Monroy E., Munoz E., Reverchon J.L. (2007). Wide bandgap UV photodetectors: A short review of devices and applications. Proc. SPIE.

[4] Liao M.Y., Sang L.W., Teraji T., Imura M., Alvarez J., Koide Y. (2012). Comprehensive investigation of single crystal diamond deep-ultraviolet detectors. Jpn. J. Appl. Phys..

[5] Toda T., Hata M., Nomura Y., Ueda Y., Sawada M., Shono M. (2004). Operation at 700 °C of 6H-SiC UV sensor fabricated using N^+^ implantation. Jpn. J. Appl. Phys..

[6] Liao M.Y., Koide Y., Alvarez J. (2005). Thermally stable visible-blind diamond photodiode using tungsten carbide Schottky contact. Appl. Phys. Lett..

[7] Munoz E. (2007). (Al,In,Ga)N-based photodetectors. Some materials issues. Phys. Stat. Sol. (b).

[8] Sang L.W., Liao M.Y., Koide Y., Sumiya M. (2011). High-temperature ultraviolet detection based on InGaN Schottky photodiodes. Appl. Phys. Lett..

[9] Zhai T.Y., Fang X.S., Liao M.Y., Xu X.J., Zeng H.B., Bando Y., Golberg D.A. (2009). Comprehensive review of one-dimensional metal-oxide nanostructure photodetectors. Sensors.

[10] Keem K., Kim H., Kim G.T., Lee J.S., Min B., Cho K., Sung M.Y., Kim S. (2004). Photocurrent in ZnO nanowires grown from Au electrodes. Appl. Phys. Lett..

[11] Soci C., Zhang A., Xiang B., Dayeh S.A., Aplin D.P.R., Park J., Bao X.Y., Lo Y.H., Wang D. (2007). ZnO nanowire UV photodetectors with high internal gain. Nano Lett..

[12] Yang Q., Guo X., Wang W., Zhang Y., Xu S., Lien D.H., Wang Z.L. (2010). Enhancing sensitivity of a single ZnO micro-/nanowire photodetector by piezo-phototronic effect. ACS Nano.

[13] Fang X.S., Bando Y., Liao M.Y., Gautam U.K., Zhi C.Y., Dierre B., Liu B.D., Zhai T.Y., Sekiguchi T., Koide Y. (2009). Single-crystalline ZnS nanobelts as ultraviolet-light sensors. Adv. Mater..

[14] Jain S.C., Narayan J., Overstraeten R.V. (2000). III-nitrides: Growth, characterization, and properties. J. Appl. Phys..

[15] Nanishi Y., Saito Y., Yamaguchi T. (2003). RF-Molecular beam epitaxy growth and properties of InN and related alloys. Jpn. J. Appl. Phys..

[16] Wu J., Haller E.E., Lu H., Schaff W.J., Saito Y., Nanishi Y. (2002). Unusual properties of the fundamental band gao of InN. Appl. Phys. Lett..

[17] Motogaito A., Ohta K., Hiramatsu K., Ohuchi Y., Tadatomo K., Hamamura Y., Fukui K. (2001). Characterization of GaN based UV-VUV detectors in the range 3.4-25 eV by using synchrotron radiation. Phys. Status Solidi (a).

[18] Pankove J.I., Berkeyheiser J.E. (1974). Properties of Zn-doped GaN. II. Photoconductivity. J. Appl. Phys..

[19] Khan M.A., Kuznia J.N., Olson D.T., van Hove J.M., Blaingame M., Reitz L.F. (1992). High-responsivity photoconductive ultraviolet sensors based on insulating single-crystal GaN epilayer. Appl. Phys. Lett..

[20] Muñoz E., Monroy E., Pau J.L., Calle F., Omnès F., Gibart P. (2001). III nitrides and UV detection. J. Phys.: Condens. Matter..

[21] Pernot C., Hirano A., Iwaya M., Detchprohm T., Amano H., Akasaki I. (1999). Low-intensity ultraviolet photodetectors based on AlGaN. Jpn. J. Appl. Phys..

[22] Jung Y.R., Lee J.H., Kim J.K., Lee Y.H., Lee M.B., Lee J.H., Hahm S.H. (2003). Pt/AlGaN metal semiconductor ultra-violet photodiodes on crack-free AlGaN layers. Jpn. J. Appl. Phys..

[23] Khan M.A., Kuznia J.N., Skogman R.A., Olson D.T., MacMillan M., Choyke W.J. (1992). Low pressure metalorganic chemical vapor deposition of AlN over sapphire substrates. Appl. Phys. Lett..

[24] Zhang J.P., Khan M.A., Sun W.H., Wang H.M., Chen C.Q., Fareed Q., Kuokstis E., Yang J.W. (2002). Pulsed atomic-layer epitaxy of ultrahigh-quality Al_x_Ga_1-x_N structures for deep ultraviolet emissions below 230 nm. Appl. Phys. Lett..

[25] Zhang J.P., Wang H.M., Gaevski M.E., Chen C.Q., Fareed Q., Yang J.W., Simin G., Khan M.A. (2002). Crack-free thick AlGaN grown on sapphire using AlN/AlGaN superlattices for strain management. Appl. Phys. Lett..

[26] Mickevicius J., Aleksiejunas R., Shur M.S., Tamulaitis G., Fareed Q., Zhang J.P., Gaska R., Khan M.A. (2005). Lifetime of nonequilibrium carriers in high-Al-content AlGaN epilayers. Phys. Status Solidi (a).

[27] Khan M.A., Shatolov M., Maruska H.P., Wang H.W., Kuokstis E. (2005). III-Nitride UV Devices. Jpn. J. Appl. Phys..

[28] Lim B.W., Chen Q.C., Yang J.Y., Khan M.A. (1996). High responsivity intrinsic photoconductors based on Al_x_Ga_1-x_N. Appl. Phys. Lett..

[29] Binet F., Duboz J.Y., Rosencher E., Scholz F., Härle V. (1996). Mechanisms of recombination in GaN photodetectors. Appl. Phys. Lett..

[30] Shen B., Yang K., Zhan L., Chen Z., Zhou Y., Chen P., Zhan R., Huang Z., Zhou H., Zheng Y. (1999). Study of photocurrent properties of GaN ultraviolet photoconductor grown on 6H-SiC substrate. Jpn. J. Appl. Phys..

[31] Monroy E., Calle F., Garrido J.A., Youinou P., Muñoz E., Omnès F., Beaumont B., Gibart P. (1999). Si-doped Al_x_Ga_1-x_N photoconductive detectors. Semicond. Sci. Technol..

[32] Qiu C.H., Pankove J.I. (1997). Deep levels and persistent photoconductivity in GaN thin film. Appl. Phys. Lett..

[33] Li J.Z., Lin J.Y., Jiang H.X., Khan M.A. (1998). Effects of persistent photoconductivity on the characteristic performance of an AlGaN/GaN heterostructure ultraviolet detector. Appl. Phys. Lett..

[34] Carrano J.C., Li T., Grudowski P.A., Eiting C.J.R., Dupuis D., Campbell J.C. (1997). High quantum efficiency metal-semiconductor-metal ultraviolet photodetectors fabricated on single-crystal GaN epitaxial layers. Electron. Lett..

[35] Carrano J.C., Li T., Brown D.L., Grudowski P.A., Eiting C.J., Dupuis R.D., Campbell J.C. (1998). Very high-speed metal-semiconductor-metal ultraviolet photodetectors fabricated on GaN. Appl. Phys. Lett..

[36] Carrano J.C., Li T., Grudowski P.A., Eiting C.J., Dupuis R.D., Campbell J.C. (1998). Comprehensive characterization of metal-semiconductor-metal ultraviolet photodetectors fabricated on single-crystal GaN. J. Appl. Phys..

[37] Li T., Lambert D.J.H., Beck A.L., Collins C.J., Yang B., Wong M.M., Chowdhury U., Dupuis R.D., Campbell J.C. (2000). Solar-blind Al_x_Ga_1-x_N-based metal-semiconductor-metal ultraviolet photodetectors. Electron. Lett..

[38] Yang B., Lambert D.J.H., Li T., Collins C.J., Wong M.M., Chowdhury U., Dupuis R.D., Campbell J.C. (2000). High-performance back-illuminated solar-blind AlGaN metal-semiconductor-metal photodetectors. Electron. Lett..

[39] Wong M.M., Chowdhury U., Collins C.J., Yang B., Denyszyn J.C., Kim K.S., Campbell J.C., Dupuis R.D. (2001). High quantum efficiency AlGaN/GaN solar-blind photodetectors grown by metalorganic chemical vapor deposition. Phys. Status Solidi (a).

[40] Jiang H., Egawa T., Ishikawa H., Shao C., Jimbo T. (2004). Visible-blind metal-semiconductor-metal photodetectors based on undoped AlGaN/GaN high electron mobility transistor. Jpn. J. Appl. Phys..

[41] Khan M.A., Kuznia J.N., Olson D.T., Blasingame M., Bhattarai A.R. (1993). Schottky barrier photodetector based on Mg-doped *p*-type GaN films. Appl. Phys. Lett..

[42] Chen Q., Yang J.W., Osinsky A., Gangopadhyay S., Lim B., Anwar M.Z., Khan M.A., Kuksenkov D., Tempkin H. (1997). Schottky barrier detectors on GaN for visible-blind ultraviolet detection. Appl. Phys. Lett..

[43] Osinsky A., Gangopadhyay S., Lim B.W., Anwar M.Z., Khan M.A., Kuksenkov D., Tempkin H. (1998). Schottky barrier photodetectors based on AlGaN. Appl. Phys. Lett..

[44] Grabowski S.P., Schneider M., Nienhaus H., Mönch W., Dimitrov R., Ambacher O., Stutzmann M. (2001). Electron affinity of Al_x_Ga_1-x_N(0001) surfaces. Appl. Phys. Lett..

[45] Chang P.C., Chen C.H., Chang S.J., Su Y.K., Yu C.L., Chen P.C., Wang C.H. (2004). AlGaN/GaN MSM photodetectors with photo-CVD annealied Ni/Au semi-transparent contacts. Semiconduct. Sci. Technol..

[46] Sang L.W., Qin Z.X., Cen L.B., Chen Z.Z., Yang Z.J., Shen B., Zhang G.Y. (2007). Barrier enhancement effect of postannealing in oxygen ambient on Ni/AlGaN Schottky contacts. Chin. Phys. Lett..

[47] Sang L.W., Qin Z.X., Cen L.B., Shen B., Zhang G.Y., Li S.P., Chen C.J., Liu D.Y., Kang J.Y., Cheng C.J. (2008). AlGaN-based solar-blind Schottky Photodetectors fabricated on AlN/Sapphire template. Chin. Phys. Lett..

[48] Biyikli N., Kimukin I., Aytur O., Ozbay E. (2004). Solar-blind AlGaN-based p-i-n photodiodes with low dark current and high detectivity. IEEE Photon. Technol. Lett..

[49] Li T., Beck A.L., Collins C., Dupuis R.D., Campbell J.C., Carrano J.C., Schurman M.J., Ferguson I.A. (1999). Improved ultraviolet quantum efficiency using a semitransparent recessed window AlGaN/GaN heterojunction p-i-n photodiode. Appl. Phys. Lett..

[50] Lambert D.J., Wong M.M., Chowdhury U., Collins C., Li T., Kwon H.K., Shelton B.S., Zhu T.G., Campbell J.C., Dupuis R.D. (2000). Back illuminated AlGaN solar-blind photodetectors. Appl. Phys. Lett..

[51] Collins C.J., Chowdhury U., Wong M.M., Yang B., Beck A.L., Dupuis R.D., Campbell J.C. (2002). Improved solar-blind external quantum efficiency of back-illuminated Al_x_Ga_1-x_N heterojunction pin photodiodes. Electron. Lett..

[52] McClintock R., Yasan A., Mayes K., Shiell D., Darvish S.R., Kung P., Razeghi M. (2004). High quantum efficiency AlGaN solar-blind p-i-n photodiodes. Appl. Phys. Lett..

[53] Su Y.K., Lee H.C., Lin J.C., Huang K.C., Lin W.J., Li T.C., Chang K.J. (2009). In0.11Ga0.89N-based p-i-n photodetector. Phys. Stat. Sol. (c).

[54] Rivera C., Pau J.L., Navarro A., Muñoz E. (2006). Photoresponse of (In,Ga)N–GaN multiple-quantum-well structures in the visible and UVA ranges. IEEE J. Quantum Electron..

[55] Rivera C., Pau J.L., Pereiro J., Munoz E. (2004). Properties of Schottky barrier photodiodes based on InGaN/GaN MQWs structures. Superlattices Microstruct..

[56] Rivera C., Pereiro J., Navarro A., Munoz E., Brandt O., Grahn H.T. (2010). Advances in Group-III-Nitride Photodetectors. Open Electr. Electron. Eng. J..

[57] Chiou Y.Z., Su Y.K., Chang S.J., Lin Y.C., Chang C.S., Chen C.H. (2002). InGaN/GaN MQW p-n junction photodetectors. Solid-State Electron..

[58] Chiou Y.Z., Su Y.K., Chang S.J., Gong J., Lin Y.C., Liu S.H., Chang C.S. (2003). High detectivity InGaN-GaN multiquantum well p-n junction photodiodes. IEEE J. Quantum Electron..

[59] Berkman E.A., El-Masry N.A., Emara A., Bedair S.M. (2008). Nearly lattice-matched n, i, and p layers for InGaN p-i-n photodiodes in the 365-500 nm spectral range. Appl. Phys. Lett..

[60] Chang P.C., Chen C.H., Chang S.J., Su Y.K., Chen P.C., Jhou Y.D., Liu C.H., Huang H., Wang S.M. (2004). InGaN/GaN multi-quantum well metal-insulator semiconductor photodetectors with photo-CVD SiO_2_ layers. Jpn. J. Appl. Phys..

[61] Chen D.J., Liu B., Lu H., Xie Z.L., Zhang R., Zheng Y.D. (2009). Improved performances of InGaN schottky photodetectors by inducing a thin insulator layer and mesa process. IEEE Electron. Dev. Lett..

[62] Chang P.C., Yu C.L. (2007). InGaN/GaN multi-quantum-well ultraviolet photosensors by capping an unactivated Mg-doped GaN layer. Appl. Phys. Lett..

[63] Yu C.L., Chuang R.W., Chang S.J., Chang P.C., Lee K.H., Lin J.C. (2007). InGaN-GaN MQW metal-semiconductor-metal photodiodes with semi-insulating Mg-doped GaN cap layers. IEEE Photon. Technol. Lett..

[64] Sang L.W., Liao M.Y., Koide Y., Sumiya M. (2011). High-performance metal-semiconductor-metal InGaN photodetectors using CaF_2_ as the insulator. Appl. Phys. Lett..

[65] Wu C.I., Kahn A. (1999). Elctronic states and effective negative electron affinity at cesiated p-GaN surfaces. J. Appl. Phys..

[66] Machuca F., Liu Z., Sun Y., Pianetta P., Spicer W.E., Pease R.F.W. (2003). Oxygen species in Cs/O activated gallium nitride (GaN) negative electron affinity photocathodes. J. Vac. Sci. Technol. B..

[67] Sumiya M., Kamo Y., Ohashi N., Takeguchi M., Heo Y.U., Yoshikawa H., Ueda S., Kobayashi K., Nihashi T., Hagino M. (2010). Fabrication and hard X-ray photoemission analysis of photocathodes with sharp solar-blind sensitivity using AlGaN films grown on Si substrates. Appl. Surf. Sci..

[68] Nemanich R.J., Baumann P.K., Benjamin M.C., English S.L., Hartman J.D., Sowers A.T., Ward B.L. (1998). Characterization of electron emitting surfaces of diamond and III-V nitrides. Diam. Films Technol..

[69] Kozawa T., Mori T., Ohwaki T., Taga Y., Sawaki N. (2000). UV photoemission study of AlGaN grown by metalorganic vapor phase epitaxy. Jpn. J. Appl. Phys..

[70] Wu C.I., Kahn A. (1999). Negative electron affinity at the Cs/AlN(0001) surface. Appl. Phys. Lett..

[71] Li D.S., Sumiya M., Fuke S., Yang D., Que D., Suzuki Y., Fukuda Y. (2001). Selective etching of GaN polar surface in potassium hydroxide solution studied by x-ray photoelectron spectroscopy. J. Appl. Phys..

[72] Razeghi M., Rogalski A. (1996). Semiconductor ultraviolet detectors. J. Appl. Phys..

[73] Kosyachenko L.A., Sklyarchuk V.M., Sklyarchuk Y.F. (1998). Electrical and photoelectric properties of Au-SiC Schottky barrier diodes. Solid-State Electron..

[74] Anikin M.M., Andreev A.N., Pyatko S.N., Savkina N.S., Strelchuk A.M., Syrkin A.L., Chelnokov V.E. (1992). UV photodetectors in 6H-SiC. Sens. Actuators A..

[75] Su Y.K., Chiou Y.Z., Chang C.S. (2002). 4H-SiC metal-semiconductor-metal ultraviolet photodetectors with Ni/ITO electrodes. Solid-State Electron..

[76] Sciuto A., Roccaforte F., Franco S.D. (2007). High efficiency 4H-SiC Schottky UV photodiodes using self-aligned semitransparent contacts. Superlattices Microstruct..

[77] Zhang Y.G., Li A.Z., Milnes A.G. (1997). Metal-semiconductor-metal ultraviolet photodetectors using 6H-SiC. IEEE Photon. Technol. Lett..

[78] Yang W.F., Zhang F., Liu Z.G., Lu Y., Wu Z.Y. (2008). High responsivity 4H-SiC based metal-semiconductor-metal ultraviolet photodetectors. Sci. China Ser. G-Phys. Mech. Astron..

[79] Edmond J., Kong H., Survorov A., Waltz D., Carter C. (2001). 6H-Silicon Carbide Light Emitting Diodes and UV Photodiodes. Phys. Status Solidi (a).

[80] Biondo S., Lazar M., Ottaviani L., Vervisch W., Le Borgne V., El Khakani M.A, Duchaine J., Milesi F., Palais O., Planson D. (2012). 4H-silicon carbide thin junction based ultraviolet photodetectors. Thin Solid Films.

[81] Bai X., Guo X., Mcintosh D., Liu H., Campbell J. (2007). High detection sensitivity of ultra violet 4H-SiC avalanche photodiodes. IEEE J. Quantum Elelctron..

[82] Liu H.D., Mcintosh D., Bai X.G., Pan H.P., Liu M.G., Campbell J.C., Cha. H.Y. (2008). 4H-SiC PIN recessed-window avalanche photodiode with high quantum efficiency. IEEE Photon. Technol. Lett..

[83] Zhu H.L., Chen X.P., Cai J.F., Wu Z.Y. (2009). 4H-SiC ultraviolet avalanche photodetectors with low breakdown voltage and high gain. Solid-State Electron..

[84] McKeag R.D., Chan S.S.M., Jackman R.B. (1995). Polycrystalline diamond photoconductive device with high UV-visible discrimination. Appl. Phys. Lett..

[85] Mohamed A.R., Lohstroh A., Sellin P.J. (2011). The effect of annealing on the X-ray induced photocurrent characteristics of CVD diamond radiation detectors with different electrical contact. Phys. Status Solidi (a).

[86] Remes Z., Izak T., Kromka A., Vanecek M. (2010). High optical quality nanocrystalline diamond with reduced non-diamond contamination. Diam. Relat. Mater..

[87] Mendoza F., Makarov V., Hidalgo A., Weiner B., Morell G. (2011). Ultraviolet photosensitivity of sulfur-doped micro- and nano-crystalline diamond. J. Appl. Phys..

[88] Smith S.D., Taylor W. (1962). Optical photon effects in the infra-red sepctrum of acceptor centres in semiconducting diamond. Proc. Phys. Soc..

[89] Koizumi S., Kamo M., Sato Y., Mita S., Sawabe A., Reznik A., Uzan-Saguy C., Kalish R. (1998). Growth and characterization of phosphorus doped *n*-type diamond thin film. Diam. Relat. Mater..

[90] Sakaguchi I., Gamo M.N., Kikuchi Y., Yasu E., Haneda H., Suzuki T., Ando T. (1999). Sulfur: A donor doped for *n*-type diamond semiconductor. Phys. Rev. B.

[91] Liao M.Y., Koide Y. (2006). High-performance metal-semiconductor-metal deep-ultraviolet photodetectors based on homoepitaxial diamond thin film. Appl. Phys. Lett..

[92] Looi H.J., Whitfield M.D., Jackman R.B. (1999). Metal-semiconductor-metal photodiodes fabricated from thin-film diamond. Appl. Phys. Lett..

[93] Liao M.Y., Alvarea J., Imura M., Koide Y. (2007). Submicron metal-semiconductor-metal diamond photodiodes toward improving the responsivity. Appl. Phys. Lett..

[94] Blank T.V., Goldberg Y.A., Kalinina E.V., Konstantinov O.V., Konstantinov A.O., Hallen A. (2005). Temperature dependence of the photoelectric conversion quantum efficiency of 4H-SiC Schottky UV photodetectors. Semicond. Sci. Technol..

[95] Adivarahan V., Simin G., Yang J.W., Lunev A., Khan M.A., Pala N., Shur M., Gaska R. (2000). SiO2-passivated lateral-geometry GaN transparent Schottky-barrier detectors. Appl. Phys. Lett..

[96] Bouhdada A., Hanzaz M., Vigue F., Faurie J.P. (2003). Electrical and optical properties of photodiodes based on ZnSe material. Appl. Phys. Lett..

[97] Liao M.Y., Koide Y., Alvarez J. (2007). Single Schottky-barrier photodiode with interdigitated-finger geometry: Application to diamond. Appl. Phys. Lett..

[98] Benmoussa A., Schuhle U., Scholze F., Kroth U., Haenen K., Saito T., Campos J., Koizumi S., Laubis C., Richter M. (2006). Radiometric characteristics of new diamond PIN photodiodes. Meas. Sci. Technol..

[99] Wang Z.L. (2004). Zinc oxide nanostructures: Growth, properties and applications. J. Phys.: Condens. Matter..

[100] Fang X.S., Bando Y., Gautam U.K., Zhai T.Y., Zeng H.B., Xu X.J., Liao M.Y., Globerg D. (2009). ZnO and ZnS nanostructures: Ultraviolet-light emitters, lasers, and sensors. Crit. Rev. Solid State Mater. Sci..

[101] Soci C., Zhang A., Bao X.Y., Kim H.K., Lo Y., Wang D.L. (2010). Nanowire photodetectors. J. Nanosci. Nanotechnol..

[102] Wang Z.L., Song J.H. (2006). Piezoelectric nanogenerators based on zinc oxide nanowire arrays. Science.

[103] Cao B.Q., Cai W.P., Sun F.Q., Zhang L.D. (2005). Ultraviolet-lightemitting ZnO nanosheets prepared by a chemical bath deposition method. Nanotechnology.

[104] Zhang L.D., Fang X.S. (2008). Controlled growth and characterization methods of semiconductor nanomaterials. J. Nanosci. Nanotechnol..

[105] Wang Z.L. (2009). ZnO nanowire and nanobelt platform for nanotechnology. Mater. Sci. Eng. R.

[106] Kind H., Yan H.Q., Messer B., Yang P.D. (2002). Nanowire ultraviolet photodetectors and optical switches. Adv. Mater..

[107] Huang J.H., Zhang K., Pan N., Gao Z.W., Wang X.P. (2008). Enhancing ultraviolet photoresponse of ZnO nanowire device by surface functionalization. Acta Phys. Sin..

[108] Lao C.S., Park M.C., Kuang Q., Deng Y.L., Sood A.K., Polla D.L., Wang Z.L. (2007). Giant enhancement in UV response of ZnO nanobelts by polymer surface-functionalization. J. Am. Chem. Soc..

[109] Mamat M.H., Khusaimi Z., Zahidi M.M., Mahmood M.R. (2011). Performance of an ultraviolet photoconductive sensor using well-aligned aluminium-doped zinc-oxide nanorod annealed in an air and oxygen enviroment. Jpn. J. Appl. Phys..

[110] Tzeng S.K., Hon M.H., Leu I.C. (2012). Improving the performance of a zinc oxide nanowire ultraviolet photodetector by adding silver nanoparticles. J. Electrochem. Soc..

[111] Calarco R., Marso M., Richter T., Aykanat A.I., Meijers R., Hart A., Stoica T., Lüth H. (2005). Size-dependent photoconductivity in MBE-grown GaN-nanowires. Nano Lett..

[112] Son M.S., Im S.I., Park Y.S., Park C.M., Kang T.W., Yoo K.H. (2006). Ultraviolet photodetector based on single GaN nanorod p-n junctions. Mater. Sci. Eng. C.

[113] Bugallo A.L., Tchernycheva M., Jacopin G., Rigutti L., Julien F.H., Chou S.T., Lin Y.T., Tseng P.H., Tu L.W. (2010). Visible-blind photodetector based on p-i-n junction GaN nanowire ensembles. Nanotechnology.

[114] Rigutti L., Tchernycheva M., Bugallo A., Jacopin G., Julien F.H., Zagonel L.F., March K., Stephan O., Kociak M., Songmuang R. (2010). Ultraviolet photodetector based on GaN/AlN quantum disks in a single nanowire. Nano Lett..

[115] Weng W.Y., Hsueh T.J., Chang S.J., Huang G.J., Hung S.C. (2011). Growth of Ga2O3 nanowires and the fabrication of solar-blind photodetector. IEEE Trans. Nanotechnol..

[116] Li L., Auer E., Liao M., Fang X., Zhai T., Gautam U., Lugstein A., Koide Y., Bando Y., Golberg D. (2011). Deep-ultraviolet solar-blind photoconductivity of individual gallium oxide nanobelts. Nanoscale.

[117] Li Y.B., Tokizono T., Liao M.Y., Zhong M., Koide Y., Yamada I., Delaunay J.J. (2010). Efficient assembly of bridged *β*-Ga_2_O_3_ nanowires for solar-blind photodetection. Adv. Funct. Mater..

[118] Feng P., Zhang J.Y., Li Q.H., Wang T.H. (2006). Individual *β*-Ga_2_O_3_ nanowires as solar-blind photodetectors. Appl. Phys. Lett..

[119] Feng P., Xue X.Y., Liu Y.G., Wan Q., Wang T.H. (2006). Achieving fast oxygen response in individual *β*-Ga_2_O_3_ nanowires by ultraviolet illumination. Appl. Phys. Lett..

[120] Tian W., Zhi C.Y., Zhai T.Y., Chen S.M., Wang X., Liao M.Y., Golberg D., Bando Y. (2012). In-doped Ga_2_O_3_ nanobelt based photodetector with high sensitivity and wide-range photoresponse. J. Mater. Chem..

[121] Fang X.S., Hu L.F., Huo K.F., Gao B., Zhao L.J., Liao M.Y., Chu P.K., Bando Y., Golberg D. (2011). New ultraviolet photodetector based on individual Nb2O5 nanobelts. Adv. Mater..

[122] Tamang R., Varghese B., Mhaisalkar S.G., Tok E.S., Sow C.H. (2011). Probing the photoresponse of individual Nb2O5 nanowires with global and localized laser beam irradiation. Nanotechnology..

[123] Wu J.M., Kuo C.H. (2009). Ultraviolet photodetectors made from SnO_2_ nanowires. Thin Solid Films.

[124] Hu L.F., Yan J., Liao M.Y., Wu L.M., Fang X.S. (2011). Ultrahigh external quantum efficiency from thin SnO_2_ nanowire ultraviolet photodetectors. Small.

[125] Tian W., Zhang C., Zhai T.Y., Li S.L., Wang X., Liao M.Y., Tsukagoshi K., Golberg D., Bando Y. (2013). Flexible SnO_2_ hollow nanosphere film based high-performance ultraviolet photodetector. Chem. Commun..

[126] Zhang D., Li C., Han S., Liu X., Tang T., Jin W., Zhou C. (2003). Ultraviolet photodetection properties of indium oxide nanowires. Appl. Phys. A.

[127] Zhang D.H., Li C., Han S., Liu X. L., Tang T., Jin W., Zhou C. W. (2003). Electronic transport studies of single-crystalline In2O3 nanowires. Appl. Phys. Lett..

[128] Li C., Zhang D.H., Han S., Liu X.L., Tang T., Zhou C.W. (2003). Diameter-controlled grown of single-crystalline In2O3 nanowires and their electronic properties. Adv. Mater..

[129] Shao D.L., Qin L.Q., Sawyer S. (2012). High responsivity, bandpass near-UV photodetector fabricated from PVA-In_2_O_3_ nanoparticles on a GaN substrate. IEEE Photon. J..

[130] Li L., Lee P.S., Yan C.Y., Zhai T.Y., Fang X.S., Liao M.Y., Koide Y., Bando Y., Golberg D. (2010). Ultrahigh-performance solar-blind photodetectors based on individual single-crystalline In_2_Ge_2_O_7_ nanobelts. Adv. Mater..

[131] Li C., Bando Y., Liao M.Y., Koide Y., Golberg D. (2010). Visible-blind deep-ultraviolet Schottky photodetector with a photocurrent gain based on individual Zn_2_GeO_4_ nanowire. Appl. Phys. Lett..

[132] Feng P., Zhang J.Y., Wan Q., Wang T.H. (2007). Photocurrent characteristics of individual ZnGa_2_O_4_ nanowires. J. Appl. Phys..

[133] Li H.G., Wu G., Chen H.Z., Wang M. (2011). Polymer/ZnO hybrid materials for near-UV sensors with wavelength selective response. Sens. Actuators B.

[134] Han Y.G., Wu G., Wang M., Chen H.Z. (2009). Hybrid ultraviolet photodetectors with high photosensitivity based on TiO_2_ nanorods array and polyfluorene. Appl. Surf. Sci..

[135] Li Y.Y., Chen C.W., Yen W.C., Su W.F., Ku C.H., Wu J.J. (2008). Near-ultraviolet photodetector based on hybrid polymer/zinc oxide nanorods by low-temperature solution processes. Appl. Phys. Lett..

[136] Zhu H., Shan C.X., Yao B., Li B.H., Zhang J.Y., Zhao D.X., Shen D.Z., Fan X.W. (2008). High spectrum selectivity ultraviolet photodetector fabricated from an n-ZnO/p-GaN heterojunction. J. Phys. Chem. C.

[137] Weng W.Y., Hsueh T.J., Chang S.J., Huang G.J., Hsueh H.T. (2011). A *β*-Ga_2_O_3_/GaN Schottky-Barrier Photodetector. IEEE Photon. Technol. Lett..

[138] Manga K.K., Wang J.Z., Lin M., Zhang J., Nesladek M., Nalla V., Ji W., Loh K.P. (2012). High-performance broadband photodetector using solution-processible PbSe-TiO_2_-Graphene hybrids. Adv. Mater..

[139] Hu L.F., Brewster M.M., Xu X.J., Tang C.C., Gradecak S., Fang X.S. (2013). Heteroepitaxial growth of GaP/ZnS nanocable with superior optoelectronic response. Nano Lett..

[140] Hu L.F., Yan J., Liao M.Y., Xiang H.X., Gong X.G., Zhang L.D., Fang X.S. (2012). An optimized ultraviolet-A light photodetector with wide-range photoresponse based on ZnS/ZnO biaxial nanobelt. Adv. Mater..

[141] Sang L.W., Hu J.Q., Zou R.J., Koide Y., Liao M.Y. (2013). Arbitary multicolor photodetection by hetero-integrated semiconductor nanostructures. Sci. Rep..

[142] Chang W.R., Fang Y.K., Ting S.F., Tsair T.S., Chang C.N., Lin C.Y., Chen S.F. (2003). The hetero-epitaxial SiCN/Si MSM photodetector for high-temperature deep-UV detecting applications. IEEE Electron Device Lett..

[143] Juang F.R., Fang Y.K., Chiang Y.T., Chou T.H., Cheng I.L. (2011). A high-performance n-i-p SiCN homojunction for low-cost and high-temperature ultraviolet detecting applications. IEEE Sens. J..

[144] Vittorio M.D., Poti B., Todaro M.T., Frassanito M.C., Pomarico A., Passaseo A., Lomascolo M., Cingolani R. (2004). High temperature characterization of GaN-based photodectors. Sens. Actuators A.

[145] Xie F., Lu H., Chen D.J., Ji X.L., Yan F., Zhang R., Zheng Y.D., Li L., Zhou J.J. (2012). Ultra-low dark current AlGaN-based solar-blind metal-semiconductor-metal photodetectors for high-temperature applications. IEEE Sens. J..

[146] Zou R.J., Hu J.Q., Sang L.W., Wu F., Zhang Z.Y., Wang C.R., Koide Y., Liao M.Y. High-detectivity high-temperature nanowires photodetectors governed by bulk photocurrent dynamics with thermally-stable carbide contacts.

